# Anxiolytic effect of antidiabetic metformin is mediated by AMPK activation in mPFC inhibitory neurons

**DOI:** 10.1038/s41380-023-02283-w

**Published:** 2023-10-05

**Authors:** Yong-mei Zhang, Hai-chao Zong, Ying-bei Qi, Liu-liu Chang, Ya-nan Gao, Ting Zhou, Tao Yin, Meng Liu, Kai-jun Pan, Wen-gang Chen, Hao-ran Guo, Fei Guo, Yan-min Peng, Min Wang, Lin-yin Feng, Yi Zang, Yang Li, Jia Li

**Affiliations:** 1 State Key Laboratory of Drug Research, Shanghai Institute of Materia Medica, Chinese Academy of Sciences, Shanghai, 201203 China; 2 School of Pharmaceutical Science and Technology, Hangzhou Institute for Advanced Study, University of Chinese Academy of Sciences, Hangzhou, Zhejiang 310024 China; 3 University of Chinese Academy of Sciences, Beijing, 100049 China; 4 Institute of Pharmaceutical Science, China Pharmaceutical University, Nanjing, Jiangsu 210009 China; 5 CAS Key Laboratory of Receptor Research, Department of Neuropharmacology, Shanghai Institute of Materia Medica, Chinese Academy of Sciences, Shanghai, 201203 China; 6 Shanghai Key Laboratory of Psychotic Disorders, Shanghai Mental Health Center, School of Medicine, Shanghai Jiao Tong University, Shanghai, 200025 China; 7 Lin Gang Laboratory, Shanghai, 200031 China; 8 National Clinical Research Center for Aging and Medicine, Huashan Hospital, Fudan University, Shanghai, 200040 China; 9 Zhongshan Institute for Drug Discovery, Shanghai Institute of Materia Medica, Chinese Academy of Sciences, Zhongshan Tsuihang New District, Zhongshan, Guangzhou 528400 China; 10 Drug Discovery Shandong Laboratory, Bohai Rim Advanced Research Institute for Drug Discovery, Yantai, Shandong 264117 China

**Keywords:** Neuroscience, Depression

## Abstract

Diabetic patients receiving the antidiabetic drug metformin have been observed to exhibit a lower prevalence of anxiety disorders, yet the precise mechanism behind this phenomenon is unclear. In our study, we found that anxiety induces a region-specific reduction in AMPK activity in the medial prefrontal cortex (mPFC). Concurrently, transgenic mice with brain-specific AMPK knockout displayed abnormal anxiety-like behaviors. Treatment with metformin or the overexpression of AMPK restored normal AMPK activity in the mPFC and mitigated social stress-induced anxiety-like behaviors. Furthermore, the specific genetic deletion of AMPK in the mPFC not only instigated anxiety in mice but also nullified the anxiolytic effects of metformin. Brain slice recordings revealed that GABAergic excitation and the resulting inhibitory inputs to mPFC pyramidal neurons were selectively diminished in stressed mice. This reduction led to an excitation-inhibition imbalance, which was effectively reversed by metformin treatment or AMPK overexpression. Moreover, the genetic deletion of AMPK in the mPFC resulted in a similar defect in GABAergic inhibitory transmission and a consequent hypo-inhibition of mPFC pyramidal neurons. We also generated a mouse model with AMPK knockout specific to GABAergic neurons. The anxiety-like behaviors in this transgenic mouse demonstrated the unique role of AMPK in the GABAergic system in relation to anxiety. Therefore, our findings suggest that the activation of AMPK in mPFC inhibitory neurons underlies the anxiolytic effects of metformin, highlighting the potential of this primary antidiabetic drug as a therapeutic option for treating anxiety disorders.

## Introduction

Fear and anxiety are adaptive, defensive responses to impending threats [1, 2]. However, when anticipatory responses become excessive or abnormal under threatening conditions, it can give rise to anxiety disorders in humans, affecting over 20% of the population at some point in their lifetime [3, 4]. As one of the most common mental health issues, anxiety disorders are typified by overwhelming anxiety, avoidance behaviors, and impairment in social and occupational functions, often resulting in significant disability [1, 2]. However, over half of patients with anxiety disorders do not respond adequately to current therapeutic treatments [4–6], underscoring the urgency for new treatment strategies.

Interestingly, type-2 diabetes patients treated with metformin, an antidiabetic drug that rectifies metabolic imbalances in peripheral tissues [7, 8], have been found to exhibit a lower incidence of anxiety disorders [9–11]. However, the underlying mechanism for this correlation remains undefined. Metformin’s potential as a treatment for neurodegenerative diseases has been investigated in animal models [12–15]. Moreover, recent studies suggest that metformin can also attenuate anxiety-like behaviors linked to several nonanxious models, such as the cerebral ischemia model, insulin-resistant model, and nicotine withdrawal model [16–18]. Nevertheless, the specific cellular and neural circuit mechanisms through which metformin influences anxiety remain to be elucidated.

Existing research indicates that metformin modifies the cellular energy state, leading to the activation of AMP-activated protein kinase (AMPK) [19], a vital cellular energy sensor crucial for preserving cellular nutrient stability [20, 21]. AMPK is instrumental in treating diseases such as diabetes and cancer [7, 22]. Recent evidence has linked AMPK to cognitive deficits seen in neurodegenerative diseases and cerebral ischemia [23, 24]. Notably, in models of stress-induced mental illness, a reduction in active Ser172-phosphorylated AMPK was observed in mouse brains [25, 26], suggesting AMPK’s significant role in anxiety. Nevertheless, our understanding of AMPK’s specific regulation and involvement in neural functions such as anxiety remains limited. Our study, utilizing repeated social defeat (RSD) mouse models and transgenic mouse models [27, 28], revealed that diminished AMPK activity in the medial prefrontal cortex (mPFC) regulates anxiety-like behavior. Moreover, we demonstrate that metformin treatment elevates AMPK activity, thereby mitigating anxiety. This research provides direct evidence linking mPFC AMPK activity with anxiety and metformin’s anxiolytic effects.

To elucidate the circuit mechanisms underpinning metformin’s anxiolytic effects, we conducted whole-cell recordings from mPFC pyramidal neurons or fluorescence-labeled GABAergic interneurons in mouse brain slices. We observed that anxiety predominantly reduces the excitability of GABAergic neurons and their inhibitory outputs to mPFC pyramidal neurons. However, this hypo-inhibition was counteracted by metformin treatment or AMPK overexpression. Combined with additional findings based on the genetic deletion of AMPK, specifically in mPFC or GABAergic neurons, or using a pharmacological inhibition method, we determined that metformin’s impact on reducing anxiety-like behaviors resulted from enhancing AMPK activity in inhibitory neurons in mPFC, thereby preventing hypoinhibition. Collectively, these insights unveil the neural circuit mechanism underlining the specific role of AMPK activity in governing the excitation–inhibition (E–I) balance in the brain and metformin’s central anxiolytic action. Our findings highlight the potential of metformin and other specific AMPK activators for treating anxiety, thereby suggesting a possible link between metabolic imbalance and mental disorders.

## Materials and methods

### Mice

All wild-type mice and transgenic mice were group-housed under standard laboratory conditions (22 ± 1 °C, 55 ± 5% humidity) with a 12:12 h light/dark schedule with food and water provided ad libitum. For all experiments with behavioral testing, male adult mice aged up to 8–10 weeks were used in the tests. All animal experiments and protocols were approved by the Animal Care and Use Committee of the Shanghai Institute of Materia Medica, where the experiments were conducted.

### Repeated social defeat (RSD)

RSD was performed as described previously with minor modifications [27, 29]. In brief, an aggressive intruder male CD-1 mouse was introduced into the cages of three established male C57BL/6 mice for 2 h per night for six consecutive nights. Submissive behaviors were checked to ensure that the resident mice showed subordinate behavior.

### Immunoblotting

Western blotting was performed to quantify protein levels in subregions of the mouse brain. All primary antibodies used for western blotting were purchased from commercial sources, as described in the Supplementary Materials and Supplementary Table.

### Drug administration

Metformin hydrochloride was diluted in 0.9% saline buffer. Mice received an intragastric injection of metformin (i.g., 250 mg/kg) once daily for 2 weeks. Vehicle was 0.9% saline administered by i.g., injection. All injections started on the day on which social stress was induced.

### Production of adeno-associated viruses (AAV)

The AAVs used here were packaged by Shanghai Taitool Bioscience Co. Ltd. (Shanghai, China) using standard methods [30], including AAV2/8-CMV_bGI-Cre-EGFP-pA, AAV2/8-CMV_bGI-EGFP-pA, AAV2/2-hSyn-mPrkaa2(T172D-TC312)-2A-mCherry, and AAV2/9-hSyn-mCherry-WPRE-pA, and viral titers were greater than 1 E + 13 particles/mL.

### Brain slice electrophysiology

Electrophysiology was performed as described previously [31]. See detailed information in the Supplementary Methods.

### Mouse behavioral tests

All behavioral tests were performed from 9:00 to 15:00 after handling for at least 3 days. Mouse movements were recorded using a video tracking system (SuperMaze video tracking software, XinRuan Information Technology, Shanghai, China). All test chambers were cleaned with 75% ethanol before and after each trial to avoid any olfactive cues.

### Quantification and statistical analysis

Most data are representative of two or three independent experiments. The data are presented as treatment means ± S.E.M.s and were analyzed by commercially available GraphPad Prism software (GraphPad Inc.). Statistical significance was defined as *P* < 0.05, and based on the results of these tests, appropriate parametric tests (two-tailed unpaired Student’s *t* test, one-way ANOVA or two-way ANOVA) were performed. No statistical methods were used to predetermine sample sizes. All datasets were tested for normalized distributions using the D’Agostino & Pearson normality test, and analytical tests were chosen accordingly.

For detailed methods, see the figure legends and Supplementary Material and Methods.

## Results

### Reduction of AMPK activity in mPFC of mouse model of anxiety disorders

**Fig. 1:**
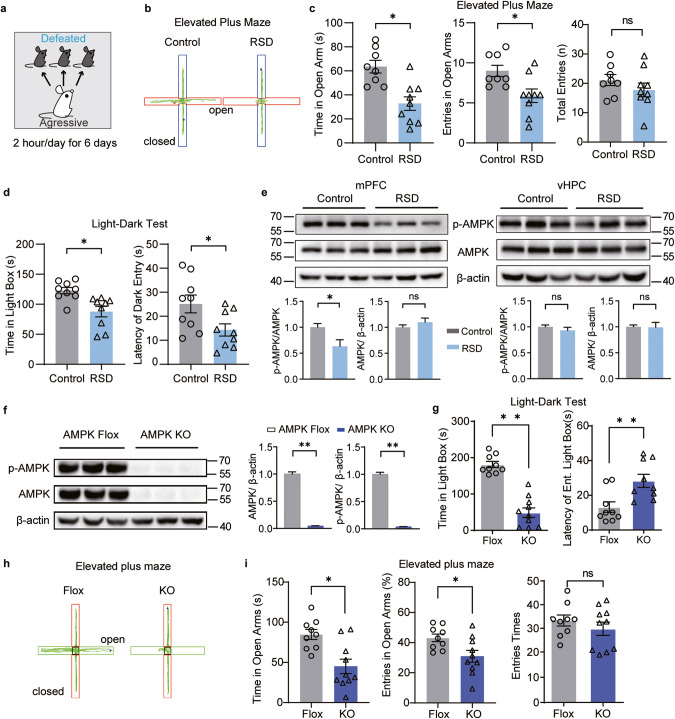
Reduced mPFC AMPK activity regulated anxiety after exposure to social stress, and genetic ablation of AMPK increased anxiety-like behaviors in mice. **a** Behavioral scheme for repeated social defeat (RSD) in C57BL/6J mice. In the EPM, (**b**) representative traces and (**c**) the time (left) and entries (middle) spent in open arms and total entries (right) in control and RSD mice. In the LDT, (**d**) the duration (left) in the light box and the latency (right) of the first time entering the dark box from the light box. **e** The expression of AMPK and p-AMPK in the mPFC and vHPC of control and RSD mice; *n* = 6–7 mice. **f** Western blotting results of AMPK KO mouse brains; *n* = 3 mice. In the LDT, (**g**) the total time that adult AMPK Flox and AMPK KO mice spent in the light box (left) and the latency of the first time to enter the light box from the dark box (right). (*n* = 9–10 mice per group). **h** The representative traces in the EPM, (**i**) the time (left), and the entries (middle) spent in the open arms of adult AMPK Flox and AMPK KO mice, the total entries shows no change between AMPK Flox and AMPK KO mice (right), *n* = 9–10 mice. Ns, not significant, *P < 0.05, **P < 0.01 by unpaired Student’s *t* test.

Psychosocial stress, a common catalyst for mood and anxiety disorders, was introduced to mice using a RSD procedure (Fig. 1a) [27, 29] to induce anxiety-like behaviors. The successful development of such behaviors was validated through four standardized behavioral tests: the elevated plus maze (EPM), light-dark test (LDT), open field test (OFT), and social interaction test (SIT). An increase in anxiety-like behavior was apparent in RSD mice, as indicated by anxiety-related parameters within these behavioral tests (Fig. 1b–d and Supplementary Fig. 1A–D).

To assess the resulting changes in brain AMPK activity, we utilized western blot analysis after sacrifice of RSD mice. We targeted two limbic brain regions for this analysis: the mPFC [32, 33] and the ventral hippocampus (vHPC) [34, 35], both of which significantly regulate anxiety in both rodent and human subjects. The analysis showed a marked decrease in the level of p-AMPK (T172 phosphorylation) in the mPFC of RSD mice compared to control mice, while no discernible difference was noted in the vHPC (Fig. 1e). This suggests that factors inducing anxiety result in a reduction in AMPK phosphorylation.

### Cerebral AMPK-deficient mice exhibited anxiety-like behaviors

To confirm the influence of AMPK on anxiety, we implemented a genetic modification to remove AMPK catalytic subunits *α1* and *α2* (AMPK KO) in mouse brains. This was achieved by crossing AMPK α1/2^loxP/loxP^ (AMPK Flox) mice with *Nestin-*Cre mice [36] (Fig. 1f). Adult AMPK KO mice demonstrated abnormal anxiety-like behaviors, as evidenced by their reduced time in the light box and showed an increased latency to the light box (Fig. 1g). Similar anxiety-like behaviors were observed in the AMPK KO mice during the EPM, as they spent less time and reduced entries in the open arms of the EPM compared to control mice (Fig. 1h, i, and Supplementary Fig. 1E). In the OFT, the AMPK KO mice spent less time in the center area and had fewer entries than the AMPK Flox mice (Supplementary Fig. 1F, G), indicating an elevated anxiety level due to AMPK deficiency. However, there was no observed difference in locomotor activity between the AMPK KO and AMPK Flox mice (Supplementary Fig. 1G). Furthermore, testing of blood glucose levels in adult AMPK KO mice revealed no significant differences compared to the AMPK Flox mice (Supplementary Fig. 1H). Collectively, these behavioral results show a marked increase in anxiety-like behaviors in AMPK KO mice.

### Metformin activation of AMPK reversed anxiety-like behaviors in RSD mice

**Fig. 2:**
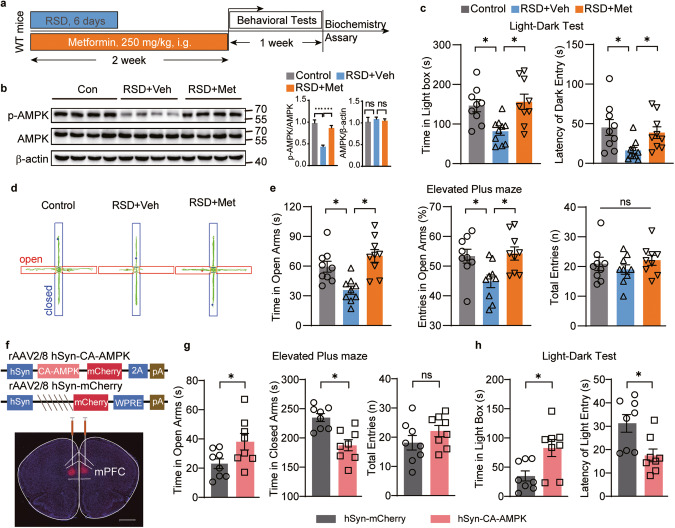
AMPK activation by metformin and overexpression of AMPK in the mPFC ameliorated stress-induced anxiety in mice. **a**–**e** RSD mice were treated with vehicle (RSD+Veh) or metformin (i.e., 250 mg/kg, RSD+Met) for 2 weeks and subsequently subjected to behavioral tests. **a** Schematic diagram of the experimental design. **b** Western blotting of mPFC tissues; *n* = 7 mice. In the LDT, (**c**) the time (left) spent in the light box and the latency (right) of mice to first enter the dark box. In the EPM, (**d**) representative traces, and (**e**) the time (left), the entries (middle) spent in the open arms, and total entries (right). In (**b,** **c,** **e**), one-way ANOVA; *n* = 9 mice. **f** Representative image of viral CA-AMPK-mCherry expression (red). Scale bar, 100 μm. In the EPM, (**g**) the time mice stayed in the open arms (left), closed arms (middle), and total entries (right) in EPM. In the LDT, (**h**) the duration mice spent in the light box (left) and the latency (right) of mice first venturing into the light box. In (**g,** **h**), unpaired Student’s *t* test, *n* = 8 mice. *P < 0.05, **P < 0.01.

Previous research indicates that in peripheral tissues, AMPK can be activated by anti-diabetic drug metformin [19, 37, 38], and that metformin reduces anxiety in diabetic patients. We sought to determine whether metformin could produce anxiolytic effects in RSD mice via AMPK-related mechanisms. Pharmacokinetic studies confirmed that metformin could traverse the blood‒brain barrier [39] following both acute (intraperitoneal, 250 mg/kg) and chronic (intragastric administration, 250 mg/kg) administration, reaching brain regions, including the mPFC and vHPC (Supplementary Fig. 2A–D). After 2 weeks of oral administration of metformin (250 mg/kg) in RSD mice, the p-AMPK in the mPFC of these mice was elevated to levels observed in control mice, while the level of AMPK expression remained consistent (Fig. 2a, b).

Next, we examined the influence of metformin on anxiety-like behaviors. In the LDT, compared to treatment with vehicle (RSD + Veh), metformin treatment (RSD + Met) increased the time spent in the light box and the latency to enter the dark box in RSD mice (Fig. 2c). In the EPM, metformin increased both the time spent in the open arm and the entries into the open arms by RSD mice (Fig. 2d, e), a pattern consistent with the anxiolytic effect of metformin. There were no differences in locomotor activity, blood glucose, or body weight between the two groups of mice (Fig. 2e, and Supplementary Fig. 2E, F, K). Metformin also significantly increased the time spent in the interaction zone and decreased the time spent in the corner zone in the SIT (Supplementary Fig. 2G–J). To further substantiate the therapeutic effect of metformin on anxiety, we administered metformin after the RSD model was established and generated a behavioral study (Supplementary Fig. 3A). Findings from the EPM and LDT revealed that metformin can alleviate anxiety-like behavior in RSD mice (Supplementary Fig. 3B, C). Taken together, our results show that metformin treatment can elevate p-AMPK in the mPFC and exert an anxiolytic effect on RSD mice. We also observed an anxiolytic effect of metformin treatment on wild-type mice (Supplementary Fig. 5A), as previously reported [40].

### Overexpression of constitutively active AMPK prevented stress-induced anxiety deficits

To assess whether overexpression of AMPK in the mPFC could counteract social stress-induced anxiety-like deficits, we utilized a viral approach. We injected AAVs expressing a constitutively active form of the *AMPK α2* subunit (CA-AMPK) [41] or vector (AAV2/9-hSyn-CA-AMPK-3xFlag-mCherry or AAV2/9-hSyn-mCherry) into bilateral mPFC neurons. The efficiency of viral expression was examined by immunofluorescence staining of mCherry and western blotting of p-AMPK, both of which confirmed expression with CA-AMPK in the mPFC (Fig. 2f and Supplementary Fig. 4A). Following RSD stress, mice overexpressing AMPK demonstrated an increase in exploratory time spent in the open arms and a decrease in time spent in the closed arms (Fig. 2g and Supplementary Fig. 4B, C). Interestingly, AMPK overexpression did not affect total entries (Fig. 2g). Furthermore, mice overexpressing AMPK exhibited a decreased latency to transition from the dark to the light box, as well as increased time spent in the light box (Fig. 2h). These outcomes from the LDT and EPM collectively support the notion that overexpression of AMPK in the mPFC is resilient to stress-induced anxiety.

### Loss of AMPK in the mPFC resulted in anxiety-like behaviors in mice

**Fig. 3:**
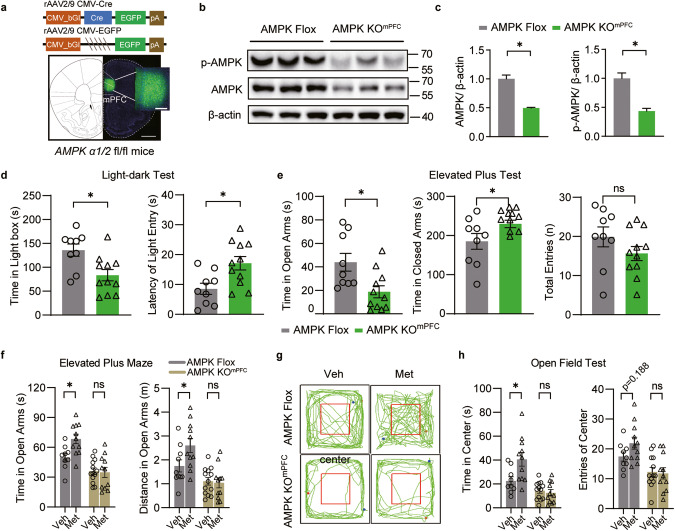
Genetic deletion of AMPK in the mPFC elicited anxiety-like behaviors and abolished the anxiolytic effect of metformin. **a** rAAV2/8 virus expressing Cre recombinase (AMPK KO^mPFC^) or EGFP control (AMPK Flox) was microinjected into the mPFC of adult AMPK Flox mice. Representative viral expression (green). Scale bar, 100 μm. **b** Western blotting of the mPFC and (**c**) related quantification; *n* = 3 mice. **d** In the LDT, the residence time in the light box (left) and the latency for mice to enter the light box from the dark box (right). **e** In EPM, the entry times in the open arms (left), the time in closed arms (middle), and the total entries in the open and closed arms (right). In (**c**, **d**, **e**), unpaired Student’s *t* test, *n* = 9 or 11 mice. **f**–**h** AMPK KO^mPFC^ and AMPK Flox mice were subjected to RSD stress and metformin treatment (i.g., 250 mg/kg) as mentioned. Veh, vehicle; Met, metformin. **f** In the EPM, the time spent (left) and the distance traveled (right) in the open arms. In the OFT, (**g**) representative traces of mice and (**h**) the time (left) and entries (right) in the center area. In (**f**, **h**), two-way ANOVA followed by Bonferroni’s test, *n* = 10–13 mice. *P < 0.05, **P < 0.01.

To further substantiate the role of mPFC AMPK in anxiety regulation, we engineered a mPFC-specific knockout mouse model (AMPK KO^mPFC^). This was achieved by microinjecting AAV2/8 expressing Cre recombinase (AAV-Cre) bilaterally into the mPFC of adult AMPK Flox mouse brains (Fig. 3a). The area of the mPFC receiving AAVs was dissected, revealing that mPFC AMPK expression levels were significantly reduced in AMPK KO^mPFC^ mice (Fig. 3b, c). Two weeks later, we conducted behavioral tests directly on AMPK KO^mPFC^ mice and control mice (AMPK Flox mice injected with AAV2/8 *CMV-*EGFP (AAV-EGFP)). In the LDT, AMPK KO^mPFC^ mice demonstrated a longer latency in transitioning from the dark box to the light box and spent less time in the light box than control mice (Fig. 3d). In the EPM, AMPK KO^mPFC^ mice spent less time in open arms and more time in closed arms, with no change in total entry times compared with control mice (Fig. 3e, and Supplementary Fig. 4D), suggesting an increase in anxiety-like behavior without locomotion changes in AMPK KO^mPFC^ mice. Similarly, anxious behaviors were also observed in AMPK KO^mPFC^ mice in the OFT (Supplementary Fig. 4E). Taken together, these behavioral results suggest that the loss of AMPK in the mPFC directly leads to anxiety-like behaviors in mice.

### mPFC-specific deletion of AMPK in the mPFC abolished the anxiolytic effect of metformin

While metformin has been shown to activate various cellular targets besides AMPK [12, 37], it is essential to ascertain the extent to which the anxiolytic effects of metformin depend on AMPK. Therefore, we subjected AMPK KO^mPFC^ mice to RSD stress and treated them with metformin. Two weeks post-AAV injection into the mPFC, anxiety-like behaviors were evaluated. Interestingly, in the EPM, AAV-EGFP-injected mice that received metformin spent more time in the open arms than vehicle-treated AAV-EGFP mice. However, metformin failed to alleviate anxiety-like behaviors in AAV-Cre mice (Fig. 3f). Similar trends were observed in the OFT parameters with respect to the time spent and entries into the center area (Fig. 3g, h), thereby implying a strong link between metformin-targetable AMPK in the mPFC and its anxiolytic effect. Consistent with our previous findings, the absence of AMPK in the mPFC also nullified the preventative effects of metformin on the development of social avoidance behavior (Supplementary Fig. 4F–H). Moreover, we found that two weeks of oral metformin administration (250 mg/kg) in AAV-EGFP-injected mice and AMPK-deficient mice resulted in an increase in p-AMPK in the mPFC of AAV-EGFP-injected mice compared to control mice. However, metformin failed to elevate the p-AMPK level in AAV-Cre mice, while the AMPK expression level remained constant in AAV-EGFP-injected mice (Supplementary Fig. 4I, J). Collectively, these results suggest that the anxiolytic effects of metformin on social stress-induced anxiety are mediated by the presence of mPFC AMPK.

### Activation of AMPK restored the excitability of GABAergic interneurons and their inhibitory outputs in the mPFC of RSD mice

**Fig. 4:**
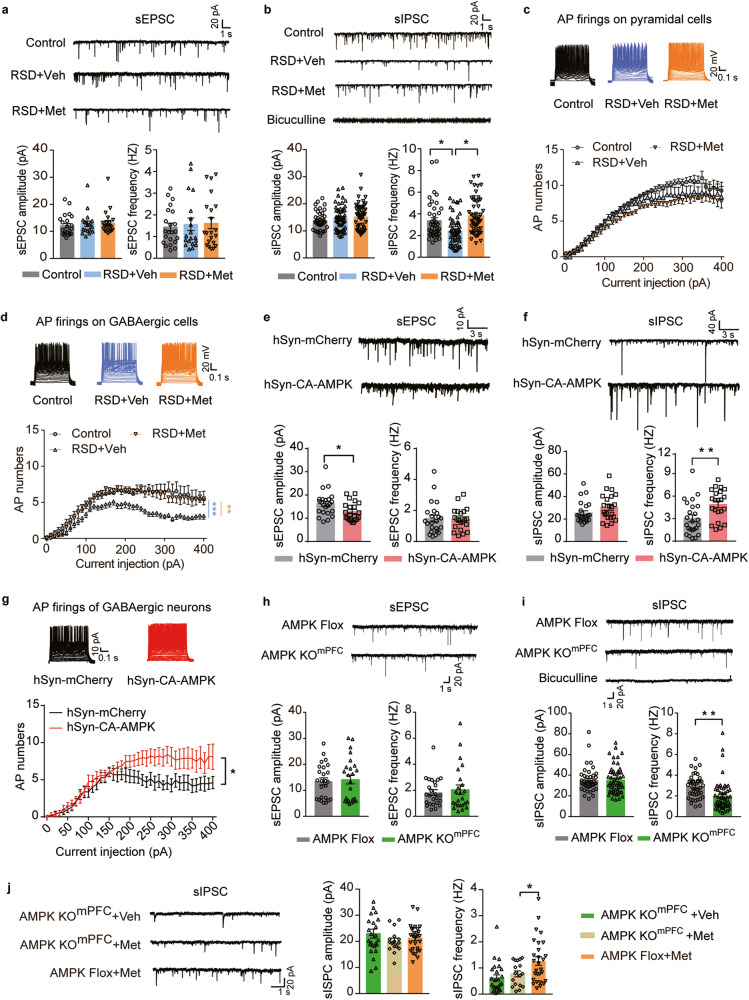
RSD induction specifically reduced mPFC GABAergic activity, and the rescue effect of oral metformin treatment was absent in AMPK KO^mPFC^ mice. **a** sEPSCs recorded from mPFC pyramidal neurons of RSD mice after metformin treatment. Example traces (top), and the statistical amplitude and the frequency (bottom), *n* = 19–23 cells from 6 mice. Veh, vehicle; Met, metformin. **b** sIPSCs recorded from mPFC pyramidal neurons of RSD mice after metformin treatment. Example traces (top) and the statistical amplitude and frequency (bottom); *n* = 41–58 cells from 6 mice. In (**a**, **b**), one-way ANOVA with Tukey’s test. **c** Action potentials (APs) from pyramidal neurons of WT mice and (**d**) from fluorescence-labeled GABAergic interneurons in *GAD1*-GFP mice subjected to RSD stress and metformin treatment, *n* = 11–18 cells from 3 to 4 mice. Two-way ANOVA followed by Tukey’s test. **e** sEPSCs recorded from mPFC pyramidal neurons of CA-AMPK mice after RSD stress. Example traces (top) and the statistical amplitude and frequency (bottom); *n* = 21–23 cells from 6 mice. **f** sIPSCs recorded from mPFC pyramidal neurons of CA-AMPK mice after RSD stress. Example traces (top) and the statistical amplitude and frequency (bottom), *n* = 20–25 cells from 6 mice. In (**e**, **f**), unpaired Student’s *t* tests were used. **g** The action potentials (APs) from GABAergic interneurons of CA-AMPK mice and AMPK-flox mice after RSD stress. All the cells patched were fluorescence-labeled GABAergic interneurons in GAD1-GFP mice subjected to virus transmission and RSD stress, n = 15–18 cells from 5 to 6 mice. **h** sEPSCs and (**i**) sIPSCs recorded from mPFC pyramidal neurons in AMPK Flox and AMPK KO^mPFC^ mice (top) and the frequency and amplitude of sEPSCs/sIPSCs (bottom). In (**e**), *n* = 24–25 cells from 3 mice; in (**f**), *n* = 43–54 cells from 3 mice; unpaired Student’s *t* test. **j** sIPSCs recorded from mPFC pyramidal neurons of AMPK KO^mPFC^ mice treated with metformin. Representative traces (left) and the amplitude and frequency of sIPSCs (right), *n* = 21–23 cells from 3 mice. One-way ANOVA with Tukey’s test. *P < 0.05; **P < 0.01.

Having established the critical role of metformin-induced AMPK activation and its influence on anxiety, we further probed the underlying circuit mechanism, particularly focusing on synaptic transmission in the mPFC. Imbalances in synaptic excitation-inhibition within the brain are widely regarded as a hallmark of anxiety [42–44]. We initiated our study by performing whole-cell patch-clamp recordings of mPFC pyramidal neurons in brain slices. Our results demonstrated a slight downward trend in spontaneous excitatory postsynaptic current (sEPSC) frequency following a 2-week oral metformin regimen in WT mice (Supplementary Fig. 5B). Remarkably, metformin substantially enhanced the frequency of spontaneous inhibitory postsynaptic currents (sIPSCs) (Supplementary Fig. 5C). Recordings from mPFC pyramidal neurons of RSD mouse brain slices revealed a notable decline in the sIPSC frequency, although no significant changes were observed in the amplitude and frequency of sEPSCs (Fig. 4a, b). Notably, metformin treatment rectified the diminished sIPSC frequency in mPFC pyramidal neurons of RSD mice (Fig. 4a, b), which implies that the social stress-induced damage to inhibitory inputs to the pyramidal neurons could be repaired with metformin treatment.

GABAergic interneurons, comprising approximately 20% of neurons within the mPFC, primarily dictate inhibitory transmission in the mPFC. To delve deeper into the alterations in GABAergic neuronal excitability, we used *GAD1*-GFP mice to label GABAergic interneurons in the mPFC [45]. We recorded the action potentials (APs) of mPFC pyramidal neurons and GFP-labeled GABAergic interneurons post RSD stress. We observed a significant impairment in the AP firing of mPFC GABAergic interneurons due to RSD, with no discernable effect on pyramidal neurons. However, metformin treatment was successful in ameliorating the downregulated AP firing of GABAergic interneurons in RSD mice (Fig. 4c, d). Notably, neither RSD nor metformin affected the membrane properties or the firing properties of pyramidal neurons and GABAergic interneurons (Supplementary Fig. 6A–L).

For a more comprehensive assessment of the alterations in synaptic transmission in mice overexpressing AMPK in the mPFC, we recorded the sEPSCs and sIPSCs of pyramidal neurons in mPFC brain slices. The recordings of sEPSCs revealed that AMPK overexpression in the mPFC markedly decreased the amplitude of sEPSCs post RSD in comparison to control mice (Fig. 4e). AMPK-overexpressing mice exhibited a significant enhancement in the sIPSC frequency of pyramidal neurons following RSD stress (Fig. 4f). We additionally recorded the APs of mPFC GABAergic interneurons in AMPK-overexpressing mice following RSD stress. The results suggested that AMPK overexpression in the mPFC significantly boosted the AP firing of GABAergic interneurons post RSD stress (Fig. 4g), with no changes observed in the membrane properties of GABAergic cells between AMPK overexpression and control mice post RSD stress (Supplementary Fig. 6M, N). Collectively, these findings underscore that AMPK overexpression in the mPFC significantly amplifies GABAergic activity compared to the control following RSD stress.

Interestingly, the amplitude and frequency of sEPSCs remained unaffected following AMPK knockout. However, the sIPSC frequency of mPFC pyramidal neurons was notably reduced in both AMPK KO mouse and AMPK KO^mPFC^ mouse brain slices (Fig. 4h, i and Supplementary Fig. 7A, B). However, metformin treatment was unable to restore the diminished sIPSC frequency in AMPK KO^mPFC^ mice (Fig. 4j). No significant alterations were observed in membrane properties or AP firing in mPFC pyramidal neurons after AMPK deletion (Supplementary Fig. 7C, D). These observations lead us to infer that anxiety decreases AMPK activity and inhibitory inputs to the mPFC pyramidal neurons in the mPFC, and this hypoinhibition could be counteracted by the AMPK-activating action of metformin.

### Inhibition of AMPK eliminated the effects of metformin on the excitability of GABAergic interneurons and their outputs to pyramidal neurons in the mPFC

**Fig. 5:**
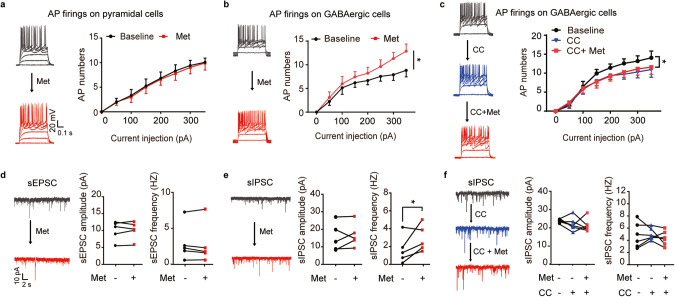
Direct effect of metformin and the requirement of AMPK activity in elevating the excitability of inhibitory interneurons and their outputs to pyramidal cells in the mPFC. **a** APs recorded from mPFC pyramidal neurons of mouse brain slices and (**b**) fluorescence-labeled GABAergic interneurons from *GAD1*-GFP mouse brain slices in the presence of ACSF (baseline) or 10 µM metformin. Representative traces of APs (left) and AP firing (right), *n* = 6–9 cells from 4 mice. **c** Representative traces of APs from GABAergic neurons in the baseline, 20 μM Compound C (CC) and 10 μM metformin perfusion, and AP firings (right), *n* = 7 cells from 3 mice. In (**a**–**c**), two-way ANOVA followed by Tukey’s post hoc test. **d** Representative traces of sEPSCs (left) from pyramidal neurons at baseline or after 10 µM metformin perfusion and the statistical amplitude (middle) and frequency (right). **e** Representative traces of sIPSCs (left) from pyramidal neurons and the statistical amplitude (middle) and frequency (right). In (**d**, **e**), a pair-paired *t* test was used. **f** Representative traces of sIPSCs (left) in baseline, CC, CC + metformin perfusion, and the statistical amplitude (middle), the frequency (right), *n* = 6–9 cells from 4 mice, one-way ANOVA followed by Dunnett’s post hoc test. *P < 0.05; **P < 0.01.

We proceeded to investigate the direct influence of metformin on the excitability of GABAergic interneurons within the mPFC. Whole-cell recording results demonstrated that incubation with metformin over 30 min did not modify the AP firing of mPFC pyramidal neurons (Fig. 5a). However, it notably increased the AP firing of GABAergic interneurons in GAD1-GFP mouse brain slices (Fig. 5b). Prior to recording, we preincubated the brain slice with an AMPK antagonist, Compound C (CC), in the recording chamber for 30 min. Our findings revealed that by suppressing AMPK activity, we could effectively neutralize metformin’s effect of increasing the AP firing of mPFC GABAergic interneurons (Fig. 5c, and Supplementary Fig. 8A, B). We also examined the direct effect of metformin on synaptic transmission ex vivo. Intriguingly, metformin incubation had no substantial impact on the sEPSCs of mPFC pyramidal neurons (Fig. 5d), but it significantly escalated the frequency of sIPSCs in comparison with the baseline (Fig. 5e). Preperfusion with CC successfully counteracted metformin’s effect of elevating the sIPSC frequency of pyramidal neurons in the mPFC (Fig. 5f). These results collectively suggest that the activation of AMPK by metformin directly amplifies the excitability of mPFC GABAergic interneurons and their inhibitory output to pyramidal neurons.

### Spontaneous anxiety-like behaviors were increased in GABAergic AMPK KO mice

We then investigated whether augmenting GABAergic transmission could ameliorate anxiety-like behavior provoked by AMPK deletion. Thirty minutes after an acute injection of muscimol (5 mg/kg, i.p.), a GABA_A_ receptor agonist [46], into AMPK KO^mPFC^ mice, we conducted a behavioral test. Our results revealed that muscimol successfully mitigated the anxiety-like behaviors of AMPK KO^mPFC^ mice in the LDT test (Supplementary Fig. 9A-B), suggesting that pharmacological enhancement of postsynaptic GABAergic transmission could alleviate anxiety caused by AMPK deficiency.

**Fig. 6:**
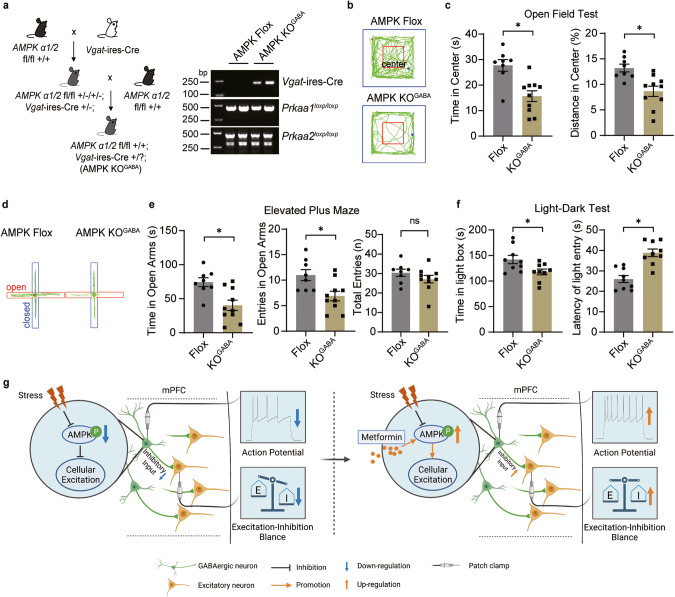
Spontaneous anxiety-like behaviors were increased in GABAergic neuron-specific AMPK knockout mice. **a** AMPK knockout in GABAergic neurons (left, AMPK KO^GABA^) and the representative result of gene validation (right). In the OFT, (**b**) representative traces and (**c**) the time (left) and distance (right) mice spent in the center area. In the EPM, (**d**) representative traces and (**e**) the duration (left) and entries (right) in the open arms. (**f**) In the LDT, time (left) spent in the light box and the latency (right) to enter the light box. **g** Schematic illustration of the AMPK-dependent anxiolytic effect of metformin by mPFC GABAergic microcircuit action. Ns, *P ˂ 0.05 by unpaired Student’s *t* test.

To further substantiate the hypothesis that heightened anxiety-like behavior is linked to AMPK dysfunction in GABAergic neurons, we generated GABAergic neuron-conditional AMPK-deleted mice (AMPK KO^GABA^) by using *Vgat*-ires-Cre mice and AMPK Flox mice (Fig. 6a and Supplementary Fig. 10A). No changes in body weight or brain mass were observed in either AMPK KO^GABA^ or AMPK Flox mice (Supplementary Fig. 10B–D). However, AMPK KO^GABA^ mice exhibited a significant escalation in anxiety-like behaviors, as indicated by the OFT (Fig. 6b, c), EPM (Fig. 6d, e), and LDT (Fig. 6f), in comparison to AMPK Flox mice, without any alterations in locomotion activity (Supplementary Fig. 10E–I). To explore the neurotransmission changes after AMPK knockout in GABAergic interneurons, we recorded the sEPSCs and sIPSCs of pyramidal neurons in mPFC slices from AMPK flox mice and AMPK KO^GABA^ mice. The results showed that AMPK knockout in GABAergic interneurons significantly increased the amplitude of sEPSCs and decreased the frequency of sIPSCs in mPFC pyramidal neurons (Supplementary Fig. 10J, K). In addition, we also tested the effect of metformin on AMPK KO^GABA^ mice. The EPM and LTD tests showed that AMPK knockout in GABAergic interneurons significantly removed the anxiolytic effect of metformin (Supplementary Fig. 11B-C), and the recordings on mPFC pyramidal neurons of AMPK KO^GABA^ mice showed that AMPK knockout in GABAergic interneurons significantly intercepted the enhancement effect of metformin on inhibitory transmission (Supplementary Fig. 10D, E). These results suggested that AMPK in GABAergic interneurons plays a crucial role in the anxiolytic effect of metformin.

In summary, our data clearly demonstrate that metformin, by modulating AMPK in mPFC GABAergic neurons, can alleviate stress-induced anxiety-like behavior.

## Discussion

Over the past decade, the inadequate response of half of the patients with anxiety to various first-line medications has highlighted the need for new pathological targets and promising novel treatments [4–6]. Repurposing conventional drugs for novel therapeutic applications might serve as a valuable strategy in the treatment of anxiety disorders [47]. Metformin, due to its efficacy, safety, and affordability, has been extensively used to regulate the blood glucose levels of patients with type-2 diabetes [7, 37, 48]. In recent years, a multitude of unexpected but beneficial effects of metformin have been discovered and confirmed for the treatment of a variety of other ailments. These include obesity, chronic liver diseases, cardiovascular disease, various forms of cancer, aging, and neurodegenerative diseases [49, 50]. Recent reports have shown that metformin produces anxiolytic effects in mouse models with various disease-related anxieties [17, 18, 24]. This study shows that metformin efficiently inhibits anxiety disorders induced by psychiatric stress, shedding light on its potential clinical usefulness for the treatment of anxiety disorders.

Metformin exhibits neuroprotective effects mainly through AMPK activation and inhibition of mitochondrial complex I. AMPK, a metabolic kinase, plays a pivotal role in maintaining cellular nutrient stabilization [20, 21]. Moreover, within the hypothalamic region of the brain, it orchestrates the regulation of the entire body’s energy balance [51–53]. In addition, AMPK is involved in regulating various cellular processes and biological functions beyond energy metabolism. Our study underlines the importance of AMPK activation for metformin’s anxiolytic effect, thereby augmenting our comprehension of AMPK’s pathological modulation in anxiety. This enhanced understanding broadens the potential clinical use of metformin for anxiolytic applications. In addition, as previously reported [25, 26], anxiety and depressive behaviors induced by psychological stress in mice coincide with diminished AMPK activity in the brain. However, the specific role of AMPK modulation in mental disorders remains controversial [16, 17, 40]. Our research provides new insights into the relationship between AMPK and anxiety. We found that AMPK phosphorylation was notably reduced in the mPFC. Elevating the phosphorylation level of AMPK using metformin induced an anxiolytic effect. Conversely, genetically removing AMPK from the mPFC directly triggered anxiety and neutralized the anxiolytic effect of metformin in mice. Therefore, we conclude that AMPK activity in the mPFC has a direct impact on the manifestation of anxiety-like behavior.

The mPFC is instrumental in stress adaptation and is a significant brain region implicated in the pathogenesis of anxiety disorders [54, 55]. Notably, an excitatory–inhibitory (E–I) imbalance in the mPFC network is present in both rodent models and humans with anxiety [42, 44, 56]. In this study, using a distress-induced mouse model and transgenic mice, we discovered that AMPK deficiency in the mPFC results in anxiety-like behaviors and impaired inhibitory synaptic transmission. Concurrently, the activation of postsynaptic GABAergic signaling by a GABA_A_ receptor agonist effectively alleviated anxiety in AMPK knockout mPFC mice. This finding suggests an impairment of presynaptic GABAergic signaling brought about by AMPK deficiency. Our results indicated that the inhibitory input to mPFC pyramidal neurons is essential for maintaining the E–I balance in the mPFC [42, 44, 56]. This E–I balance was disrupted by AMPK deficiency but restored with metformin treatment.

GABAergic interneurons, constituting approximately 20% of neurons within the prefrontal cortex, are known to regulate anxiety in both patients and stress-affected animals [42, 44, 57, 58]. Nevertheless, tangible evidence identifying unique characteristics of AMPK in GABAergic neurons remains elusive. Our electrophysiological findings revealed that metformin-induced AMPK activation preferentially heightened the excitability of GABAergic interneurons and their inhibitory outputs to mPFC pyramidal neurons without directly impacting pyramidal neurons. Fast-spiking interneurons, which generate APs at high frequencies, demand a high level of energy expenditure reliant on oxidative phosphorylation for ATP generation [59–61]. This energy requirement may explain the efficient response of mPFC GABAergic interneurons to metformin-activated AMPK. In our efforts to further validate the modulation of AMPK in GABAergic neurons on anxiety, we developed a mouse line in which AMPK was specifically deleted in GABAergic neurons. These transgenic mice displayed abnormal anxiety-like behaviors, suggesting a crucial role of AMPK in inhibitory neuronal transmission in anxiety. Due to technical constraints, we could not create a mouse line with a specific deletion of AMPK in mPFC GABAergic interneurons. However, it is worth noting that AMPK activation might specifically enhance the firing activity of inhibitory neurons through varied mechanisms, potentially involving different protein synthesis or ion channels activated by AMPK. Several studies have found that the downstream pathway of AMPK can regulate the activities of many ion channels, including Kv2.1, Kv7.1, K2P2.1 (TREK1), K2P9.1 (TASK-3), and K2P10 (Tre-2) [62–66]. In addition, AMPK may also regulate some ion channels that are specifically expressed in GABAergic interneurons, such as Nav1.1, Kv3.1, and Kv3.2 [67, 68]. Therefore, the channels regulating the firing of GABAergic interneurons warrant further investigation.

In summary, our results from both loss- and gain-of-function experiments robustly support the hypothesis that AMPK dysfunction leading to impaired GABAergic transmission in the mPFC induces anxiety-like behaviors. The activation of AMPK by metformin or other pharmacological agonizts, aiming to restore inhibitory transmission, might represent a promising therapeutic strategy for treating anxiety disorders. This can be evaluated in future clinical trials.

### Supplementary information

Supplemental material

Supplemental figure 1

Supplemental figure 2

Supplemental figure 3

Supplemental figure 4

Supplemental figure 5

Supplemental figure 6

Supplemental figure 7

Supplemental figure 8

Supplemental figure 9

Supplemental figure 10

Supplemental figure 11

Key Resources Table
