# Supplementary material for: Anxiolytic effect of antidiabetic metformin is mediated by AMPK activation in mPFC inhibitory neurons
Source: Mol Psychiatry. 2023 Oct 5;28(9):3955–65. doi: 10.1038/s41380-023-02283-w (PMC10730396; doi:10.1038/s41380-023-02283-w)
Supplement: Supplementary file 1 — Supplemental material [file 41380_2023_2283_MOESM1_ESM.docx]

**Supplementary Information for**

**Anxiolytic effect of antidiabetic metformin is mediated by AMPK activation in mPFC inhibitory neurons**

**The PDF file includes supplementary Methods and Materials, 8 Supplementary figures, and related captions.**

**Authors:**

Yong-mei Zhang^1,2,3^, Hai-chao Zong^1,3^, Ying-bei Qi^1,2,3^, Liu-liu Chang^1,3^, Ya-nan Gao^1,4^, Ting Zhou^1,3^, Tao Yin^1,3^, Meng Liu^1,3^, Kai-jun Pan^1,4^, Wen-gang Chen^1,4^, Hao-ran Guo^1,3^, Fei Guo^3,5^, Yan-min Peng^1,6^, Min Wang^1,3^, Lin-yin Feng^3,5^, Yi Zang^1,3,7^, Yang Li^3,5,8^, Jia Li^1,2,3,4,9,10^

**Materials and methods**

**Mice.**

All C57BL/J6 mice were group housed (four mice per cage in the different experiments) under standard laboratory conditions (22 ± 1 °C, 55 ± 5% humidity) with a 12:12 h light/dark schedule with food and water provided ad libitum. Twelve-month-old male CD-1 mice (retired breeders) were used in the RSD model and fed alone. Eight- to ten-week-old wild-type male mice were used for the stress model and metformin treatment experiments. AMPK α1/2 double flox mice were generated from Prkaa1loxp/loxp mice and Prkaa2 loxp/loxp mice (The Jackson Laboratory, Stock NO:014141, and NO:014142), which were two catalytic subunit genes of AMPKα. AMPK KO mice were generated by using AMPK α1/2 double flox mice and Nestin-Cre mice (The Jackson Laboratory, Stock NO: 003771), and GABAergic neuron-specific AMPK knockout mice were generated by using AMPK α1/2 double flox mice and Vgat-ires-Cre mice (The Jackson Laboratory, Stock NO: 016962). GAD1-GFP mice (The Jackson Laboratory, Stock NO: 007677) were a gift from ShanghaiTech University. For all experiments with behavioral testing, knockout and flox male adult mice were from the same pregnant mice and grew up to 8-10 weeks before testing. All animal experiments and protocols were approved by the Animal Care and Use Committee of the Shanghai Institute of Materia Medica, where the experiments were conducted.

**Repeated social defeat (RSD).**

RSD was performed as described previously with minor modifications26,28. Briefly, an aggressive male intruder CD-1 mouse was introduced into cages of three established male cohorts of C57 mice for 2 hours between 17:00 and 19:00 for six consecutive nights. During each cycle, submissive behaviors (e.g., upright posture, fleeing, and crouching) were observed to ensure defeat of the C57 mice. A new intruder was introduced if CD1 mice did not initiate an attack on the resident mice within the first 10 minutes or if it was defeated by any of the resident mice. At the end of the 2 h period, the intruder was removed, and the residents were left undisturbed until the following day the paradigm was repeated. To avoid habituation, different intruders were used on consecutive nights. The health status of the mice was carefully examined throughout all experiments. Mice that were injured or moribund were removed from the study. In the end, less than 10% of mice met the early removal criteria. Control mice were left undisturbed in their home cages. All behavior and biological measures were obtained 14 h after the final cycle.

**Production of adeno-associated viruses (AAV).**

The AAVs used here were packaged by Shanghai Taitool Bioscience Co., Ltd. (Shanghai, China) using standard methods29, including AAV2/8-CMV_bGI-Cre-EGFP-pA, AAV2/8-CMV_bGI-EGFP-pA, AAV2/2-hSyn-mPrkaa2(T172D-TC312)-2A-mCherry, and AAV2/9-hSyn-mCherry-WPRE-pA, and viral titers were greater than 1E+13 particles/mL. The constitutively active AMPKα2 cDNA construct was designed and constructed according to a previous study30, which encodes residues 1-312 of AMPK α2 mutated on the threonine 172 residue to an aspartic acid (T172D).

**Stereotactic injections.**

WT mice or AMPKα1/2 flox mice (8-10 weeks) were anesthetized with inhalation of isoflurane, 2% v/v in O2, and placed in a stereotaxic apparatus (RWD, Shenzhen, China). A midline incision in the skin was made using a cleaned blade to expose the skull to identify bregma and lambda. Mice were injected bilaterally with approximately 0.3 μL of AAVs, 2E+12 infection units per mL, into the dmPFC (coordinates from bregma: +1.9 mm anterior/posterior, ±0.32 mm medial/lateral, －1.75 mm dorsal/ventral) using glass microelectrodes at a slow rate (100 nL per min). Viral expression of Cre or AMPKα is dependent on different experiments. The injection microelectrode was kept at the site for 5 min after the virus infusion and slowly withdrawn. Viral expression was assessed 2 weeks after surgery.

**Western blot.**

All primary antibodies used for western blots were purchased from commercial sources. AMPK (CST, 1:1000), p-AMPK (CST, 1:1000), and anti-β-actin (Abcepta, 1:5000). The mPFC and vHPC regions were quickly dissected from mice after sacrifice by using brain matrices. Tissue samples were homogenized in cold RIPA lysis buffer (P0013B, Beyotime) supplied with protease and phosphatase inhibitors (P1006, Beyotime) and lysed for 30 min. Lysates were cleared by centrifugation at 12,000 rpm (15,294 × g) for 30 min. Protein concentration was determined using a standard BSA method, and 30 µg of total protein for each sample was separated by SDS‒PAGE followed by Western blot analysis. After electrophoresis, separated proteins were transferred onto polyvinylidene difluoride membranes (SLGP033RB, Millipore). For immunoblotting, the membrane was blocked with 5% nonfat milk. After incubation with specific primary antibody overnight, horseradish peroxidase-conjugated secondary antibody was applied. The positive immune reactive signal was detected by using ECL (RPN2236, GE Healthcare).

**Patch-clamp recording.**

Preparing for the acute slices: the mice were anesthetized with pentobarbital sodium (80 mg/kg, i.p.) and then perfused with 20-40 mL of ice-cold and preoxygenated artificial cerebrospinal fluid (ACSF) containing (in mM): 120 NaCl, 2.5 KCl, 26 NaHCO3, 1 NaH2PO4, 10 D-glucose, 1.3 MgCl2 and 2.5 CaCl2, gassed with 95% O2 and 5% CO2. The brain was immediately dissected into 300 μm-thick slices containing the mPFC after decapitation using a vibratome (Leica VT1000s, USA). Then, mPFC slices were quickly transferred to the recovery chamber, incubated in normal ACSF at 28°C for 1 h and gassed with 95% O2 and 5% CO2. Finally, the slices were transferred to the recording dish at room temperature for recording.

Electrophysiology recording: All recordings on mPFC slices were performed using a Multiclamp 700 B amplifier (Molecular Device, USA) under an Olympus microscope (Olympus, Japan) equipped with infrared differential interference contrast optics. Briefly, recordings were performed in ACSF, gassed with 95% O2 and 5% CO2. All the resistances of the glass electrodes were controlled in the range of 3-8 MΩ. The intracellular solution for sEPSCs recording contained (in mM): 115 CsMeSO3, 20 CsCl, 10 HEPES, 2.5 MgCl2, 4 Na2-ATP, 0.4 Na-GTP, 10 Na-phosphocreatine, and 0.6 EGTA (pH 7.35). The intracellular solution for sIPSCs recording contained (in mM) 110 CsCl, 30 K-Gluconic acid, 0.1 CaCl2, 10 HEPES, 4 Mg-ATP, 0.3 GTP, and 1.1 EDTA, and the pH was adjusted to 7.3 with KOH. Bicuculline (10 μmol/mL) was added to the perfusing solution to block GABAA-mediated sISPSCs. For current clamp recordings, electrodes were filled with an internal solution containing the following (in mM): 130 K-gluconate, 4 KCl, 10 HEPES, 5 Na2-phosphocreatine, 0.1 EGTA, 4 Mg-ATP, and 1 GTP (pH 7.2). For recording sEPSCs at -60 mV and for recording sIPSCs at -80 mV, CNQX (20 μM) and MK-801 (10 μM) were added to ACSF. Membrane currents (voltage-clamp) were acquired at a 50 kHz sampling frequency using a Digidata 1440A (Molecular Devices, USA) after low-pass filtering at 2 kHz with the Bessel filter built into the EPC-10 amplifier.

**Electrophysiology and data analysis.**

The sEPSC and sIPSC data were analyzed by the Mini Analysis Program (Synaptosoft). The current clamp recording data were analyzed by clampfit. Resting membrane potential (RMP) was the membrane voltage measured in current clamp mode immediately after reaching the whole-cell configuration. Input resistance (Rin) was calculated as the slope of the linear fit of the voltage-current plot (I-V curve) generated from a family of hyperpolarizing and depolarizing current injections (−60 to +10 pA, steps of 10 pA). AP firing output was calculated as the number of action potentials (APs) fired in response to 500 ms long depolarizing current injections (0–400 pA, steps of 10 pA). Rheobase was measured as the minimum current injection that elicited spiking. Firing traces in response to a 50 pA current above the rheobase were used for analysis of single AP properties, including AP threshold, AP amplitude, AP half-width and fast after hyperpolarization (fAHP) amplitude. The threshold was defined as the voltage at which the value of the third derivative of the voltage with time was maximum. Action potential amplitude was measured from threshold to peak, with the half-width measured at half this distance. Fast after hyperpolarization was measured from the threshold to the negative voltage peak after the AP.

**Mouse behavioral tests.**

All behavioral tests were performed from 9:00-15:00 after handling for at least 3 days. Mouse movement was recorded using a video tracking system (SuperMaze video tracking software, XinRuan Information Technology, Shanghai, China). All test chambers were cleaned with 75% ethanol before and after each trial to avoid any olfactive cues.

The **elevated plus maze** has two open arms (35×6 cm), two closed arms (35×6×15 cm), and a central platform (5×5 cm). The maze is 40 cm elevated above the ground. Mice were individually placed in the central platform and head to open arms and allowed to explore the maze for 5 minutes. The **open field test** is a behavioral assay that is widely used to measure anxiety-like behaviors and locomotion behaviors. We used a square box (40×40×40 cm) and placed mice in one corner facing the center area. Mice were allowed to freely move for 5 minutes, and data were videotaped and analyzed. The **light-dark test** is performed based on the innate aversion of mice to brightly illuminated areas. The test apparatus contains a light zone (27×27×30 cm) and a dark zone (18×27×30 cm). The dark zone is enclosed and has significantly less light (<3 lux). Anxious mice entered the dark zone faster and spent more time in the dark zone in a 5-minute recording. The **social interaction test** was performed as previously described with little modification 31. In brief, in Trial 1 (single), an experimental mouse was placed into the arena with an empty columnar wire mesh cage, and activity was recorded for 2.5 minutes. In trial 2 (pair), an unfamiliar CD-1 mouse was placed in the wire mesh cage, the experimental mouse was placed in the arena again, and activity was recorded for another 2.5 min. The activity in the social avoidance behavior test was video recorded and analyzed.

**Quantification and statistical analysis**

All data are representative of two or three independent experiments. All data are presented as treatment means ± S.E.M.s and were analyzed by commercially available GraphPad Prism software (GraphPad Inc.). Statistical significance was defined as P < 0.05, and based on the results of these tests, appropriate parametric tests (two-tailed unpaired Student’s t test, one-way ANOVA or two-way ANOVA) were performed. No statistical methods were used to predetermine sample sizes. All datasets were tested for normalized distributions using the D’Agostino & Pearson normality test, and analytical tests were chosen accordingly.

**Supplemental figures**

**Fig. S1. Adult AMPK KO mice exhibited anxiety-like behaviors and had no locomotor alterations.**

(**A**) Representative traces in the SIT of control and RSD mice and (**B**) the duration (left) and SI ratio (right) in the interaction zone. IZ, interaction zone; CZ, corner zone. (**C**) The entries in open arms (%) in EPM. (**D**) In the OFT, time spent in the center (left), entries into the center (middle), and total distance traveled (right) during a 5 min test of adult AMPK Flox and AMPK KO mice. In the EPM test, **(E)** travel distance in the open arms of adult AMPK Flox and AMPK KO mice. (**F**) Representative traces in the OFT, and (**G**) the duration (left), number of entries (middle) of the center area, and the total distance moved (right). (**H**) Blood glucose levels in AMPK Flox and AMPK KO mice. Data represent means ± SEMs; ns, not significant, *P < 0.05, *P < 0.01 by unpaired Student’s t test.

**Fig. S2. Pharmacokinetic studies of metformin and its anxiolytic effect. (A to D)** Pharmacokinetic studies of acute injection of metformin (i.p., 250 mg/kg) or chronic injection of metformin (i.g., 250 mg/kg per day) 2 weeks in wild-type mice. The mean concentrations of metformin in the **(A and B)** plasma, **(C and D)** mPFC and vHPC are shown at various time points. (n=3-4 mice at each time point). **(E)** Blood glucose levels and **(F)** body weight changes in stressed mice treated with metformin or vehicle were monitored daily. In the social interaction test, **(G)** the time spent in the interaction zone, **(H)** the social interaction ratio (pair/single) of mice spent in the interaction zone, **(I)** the time spent in the corner zone, and **(J)** the social interaction ratio (pair/single) of mice spent in the corner zone. (**K**) In the OFT, the time (left) and distance (middle) spent in the center area and the total distance moved (right). Data represent means ± S.E.M.s ns, not significant, *P < 0.05, **P < 0.01 by two-way ANOVA followed by Dunnett’s pairwise comparisons.

**Fig S3. Metformin administration after modeling significantly relieved anxiety-like behavior in the RSD group. A**. Metformin administration after modeling reversed the behavior of the RSD group in the elevated plus maze test, entries (left) and time (right) in open arms, n=9 mice. **B.** Metformin administration after modeling reversed the behavior of the RSD group in the light-dark test, the latency of light entry (left) and time in the light box (right). N=9~10 mice, statistics are measured by one-way ANOVA followed by Dunnett's post hoc test. *P < 0.05; **P < 0.01.

**Fig. S4. Genetic modulation of AMPK in the mPFC had no impact on locomotion activity and regulated anxiety-like behaviors in mice. (A)** Western blotting of AMPK (30 kd), ULK1, and p-ULK1 in the mPFC (left) and the related quantification (right) of mCherry-expressing control mice and CA-AMPK-overexpressing mice. **(B-C)** Supplemental data from the elevated plus maze of mCherry-expressing control mice and CA-AMPK-overexpressing mice related to Fig. 2, n=8 mice. **(D)** The elevated plus maze results of AMPK Flox and AMPK KO^mPFC^ related to Fig. 3, n=9~10 mice. (**E**) In the OFT, the time (left) and distance (middle) spent in the center and the locomotion activity (right) of AMPK Flox and AMPK KO^mPFC^ mice. **(F-H)** AMPK Flox and AMPK KO^mPFC^ mice were subjected to repeated social defeat and treated with metformin. **(F)** Representative traces of different groups of mice in the social interaction test when targeted CD1 was placed into the interaction zone. Interaction zone, IZ; corner zone, CZ. **(G)** Social interaction (SI) ratio of IZ (paired/single). **(H)** Social interaction (SI) ratio of CZ (paired/single). N=7 mice. (**I**) Western blotting of mPFC tissues from AMPK-Flox mice and AMPK-KO^mPFC^ mice and all mice separately treated with vehicle and metformin. (**J**) Quantified expression of p-AMPK (left) and AMPK (right) in the four groups by densitometry, n= 6 mice. In C to E, unpaired Student’s t test was used. In G and H, one-way ANOVA was used. in J Statistics were measured by one-way ANOVA followed by Dunnett's post hoc test (B). Data represent means ± S.E.M.s ns, not significant, *P < 0.05, **P < 0.01，***P < 0.01.

**Fig. S5.** **Metformin treatment for two weeks induced anxiolytic effects and significantly increased inhibitory output to pyramidal neurons**. (**A**) WT mice were administered metformin (i.g., 250 mg·kg-1) for 2 weeks and evaluated in the OFT. The time (left) and distance (right) traveled in the center (n=10, unpaired Student’s t test, *P < 0.05). **(B-C)** Example traces of sEPSCs (B-top) and sIPSCs (C-top) were recorded from mPFC pyramidal neurons of WT and metformin-treated mice. Comparison of the amplitude and frequency of sEPSCs and sIPSCs showed that metformin treatment significantly increased the sIPSC frequency of pyramidal neurons (traces were recorded from 14–42 cells from 3-4 biologically independent mice per group, one-way ANOVA with Tukey’s test). Data represent means ± S.E.M.s ns, not significant, *P < 0.05 by unpaired Student’s t test. *P < 0.05; **P < 0.01.

**Fig. S6. Metformin showed no effect on the cell properties of mPFC pyramidal neurons and GABAergic interneurons**. **(A-F)** Cell properties recorded from mPFC pyramidal neurons. **(A)** Example voltage traces in response to subthreshold current injections from -60 pA to + 10 pA in WTs (black), RSD (blue) and metformin-treated mice (orange). Comparison of input resistance injections (R_in_) (**B**) and resting membrane potential (RMP) (**C**) (one-way ANOVA). (**D**) Rheobase of pyramidal cells. (**E**) Comparison of AP amplitude. (**F**) AP threshold. WTs, n=15 cells, 3 mice; RSD, n=11 cells, 3 mice; Met, n=18 cells, 4 mice. One-way ANOVA test. **(G-I)** Properties of action potentials recorded from mPFC GABAergic interneurons. **(G)** Example voltage traces in response to subthreshold current injections from -60 pA to + 10 pA in WTs (black), RSD (blue) and metformin-treated mice (orange). Comparison of input resistance injections (R_in_) (**H**) and resting membrane potential (RMP) (**I**), one-way ANOVA test. Rheobase of GABAergic cells (**J**), Comparison of AP amplitude (**K**) and AP threshold **(I)** from GABAergic interneurons in the mPFC. WTs, n=21 cells, 3 mice; RSD, n=24 cells, 3 mice; Met, n=19 cells, 4 mice; one-way ANOVA. (**M-N**) Action potentials (APs) from GABAergic interneurons of CA-AMPK mice and AMPK-flox mice after RSD stress, n = 15-18 cells from 5~6 mice. Data are presented as the mean ± S.E.M. *p< 0.05; **p< 0.01, ***p< 0.001.

**Fig. S7. Central neural AMPK-deleted mice showed increased anxiety-like and decreased inhibitory output to pyramidal neurons in the mPFC.** (**A-B**) Example traces showing sEPSCs (a-left) and sIPSCs (b-left) recorded from pyramidal neurons in flox and AMPK KO mice. Comparison of the amplitude and frequency of sEPSCs (**A**) and sIPSCs (**B**) showed that global deletion of AMPK significantly decreased the sIPSC frequency of pyramidal neurons (traces were recorded from 14–42 cells from 3-4 biologically independent mice per group, one-way ANOVA with Tukey’s test). **(C)** Example voltage traces in response to subthreshold current injections from -60 pA to +10 pA in Flox (dark gray) and AMPK KO (blue) (left). Comparison of resting membrane potential (RMP) (AMPK Flox vs AMPK KO, p=0.163) and input resistance injections (R_in_). **(D)** The action potential of the mPFC pyramidal neurons exhibited no changes after AMPK deletion. ⅰ, example traces in response to suprathreshold current injections of 200 pA and 400 pA. ⅱ, number of APs fired (two-way ANOVA with Sidak’s test, Flox vs AMPK KO (F_1,1886_=0.2984, p=0.5849)); ⅲ, Rheobase of Flox and AMPK KO ((Flox vs KO, p=0.2719), unpaired two-tailed t test); ⅳ, comparison of AP amplitude (p=0.6393, unpaired two-tailed t test); ⅴ, AP threshold (p=0.7780, unpaired Student’s t test). Flox, n=27 cells, 3 mice; AMPK KO, n=26 cells, 3 mice. Data are presented as the mean ± S.E.M. *p<0.05, **p<0.01, ***p<0.001.

**Fig. S8. AMPK inhibitor (compound C, CC) incubation significantly decreased the sIPSCs of pyramidal neurons and the AP firing of GABAergic interneurons. (A)**. CC treatment markedly inhibited the frequency and amplitude of sIPSCs on pyramidal neurons in mPFC slices (n= 5 cells from 3 mice.). One-way ANOVA followed by Dunnett's post hoc test. (**B)**. CC treatment significantly inhibited the AP firing numbers of GABAergic interneurons in mPFC slices (n=7). Two-way ANOVA followed by Tukey's post hoc test. *P < 0.05; **P < 0.01, ***P < 0.001.

**Fig. S9. The GABA_A_ receptor agonist muscimol reversed anxiety-like behaviors in mPFC-specific AMPK-deficient mice.** *AMPK α1/2* floxed mice were microinjected with AAVs expressing Cre recombinase in the mPFC. Two weeks later, these mice were treated with a single injection of muscimol (i.p., 10 mg/kg), a GABA_A_ receptor agonist, after the mice recovered from surgery. Then, the LDT was used to test the anxiety level of the mice 30 mins later. **(A)** The duration that the experimental group spent in the light box and **(B)** the latency of mice to first enter the dark box from the light box (n=9 mice per group). Data represent means ± S.E.M.s ns, not significant, *P < 0.05, **P < 0.01 by one-way ANOVA.

**Fig. S10. Specific ablation of AMPK in GABAergic neurons resulted in anxiety-like behaviors and decreased inhibitory transmission.** **(A)** Immunohistofluorescence analysis of AMPK (red) and GAD1/2 (green) in the mPFC of AMPK-Flox mice and Vgat-AMPK-KO mice. Scale bar 10 μm (left). Higher-magnification views of the boxed regions in the fluorescence images are shown on the right. Scale bars, 5 μm (right). n= 6 mice**. (B)** Body weight of adult mice at 8-10 weeks (n=5-7 mice per group). **(C)** Representative image of adult AMPK Flox and AMPK KO^GABA^ mouse brains and **(D)** the calculated brain weight (n=3-4 mouse brains per group). In the OFT, **(E)** the entry times in the center of open field, and **(F)** the travel distance in the center, and **(G)** the total movement during 5 min recording of adult AMPK Flox and AMPK KO^GABA^ mice. In the EPM, **(H)** the entries in the closed arms and **(I)** the entries in the open arms during 5 min recording in the plus maze. (n=8-10 mice per group). **(J)**. sEPSCs recorded from mPFC pyramidal neurons of AMPK flox and AMPK KO^GABA^ mice. Example traces of two groups (left), and the statistical amplitude (middle) and the frequency (right), n = 26~39 cells from 6 mice. Flox: AMPK flox mice, KO^GABA^: KO^GABA^ mice. **(K)**. sIPSCs recorded from mPFC pyramidal neurons of AMPK flox and AMPK KO^GABA^ mice. Example traces of two groups (left), and the statistical amplitude (middle) and the frequency (right), n = 22-38 cells from 6 mice. Flox: AMPK flox mice, KO^GABA^: KO^GABA^ mice. Data represent means ± S.E.M.s ns, not significant, *P < 0.05, **P < 0.01 by unpaired Student’s t test.

**Fig. S11. AMPK knockout in GABAergic interneurons significantly removed the metformin-induced anxiolytic effect and enhanced GABAergic activity.** **(A)**. Water consumption of mice. **(B)**. In the EPM, there was no difference between the vehicle and metformin treatment groups of AMPK KO^GABA^ mice in entries in open arms, the time in open and total entries. **(C)**. In the LDT, the time spent in the light box and the latency to dark were not different between the vehicle and metformin groups of AMPK KO^GABA^ mice. **(D)**. sEPSCs recorded from mPFC pyramidal neurons of vehicle and metformin groups in AMPK KO^GABA^ mice. Example traces of two groups (left), and the statistical amplitude (middle) and the frequency (right), n = 23-26 cells from 6 mice. **(E)**. sIPSCs recorded from mPFC pyramidal neurons of vehicle and metformin groups in AMPK KO^GABA^ mice. Example traces of two groups (left), and the statistical amplitude (middle) and the frequency (right), n = 19-29 cells from 6 mice. Data represent means ± S.E.M.s ns, not significant, *P < 0.05, **P < 0.01 by unpaired Student’s t test.

**Supplementary information is available at MP’s website**
