# Supplementary figures and images for: Anxiolytic effect of antidiabetic metformin is mediated by AMPK activation in mPFC inhibitory neurons
Source: Mol Psychiatry. 2023 Oct 5;28(9):3955–65. doi: 10.1038/s41380-023-02283-w (PMC10730396; doi:10.1038/s41380-023-02283-w)

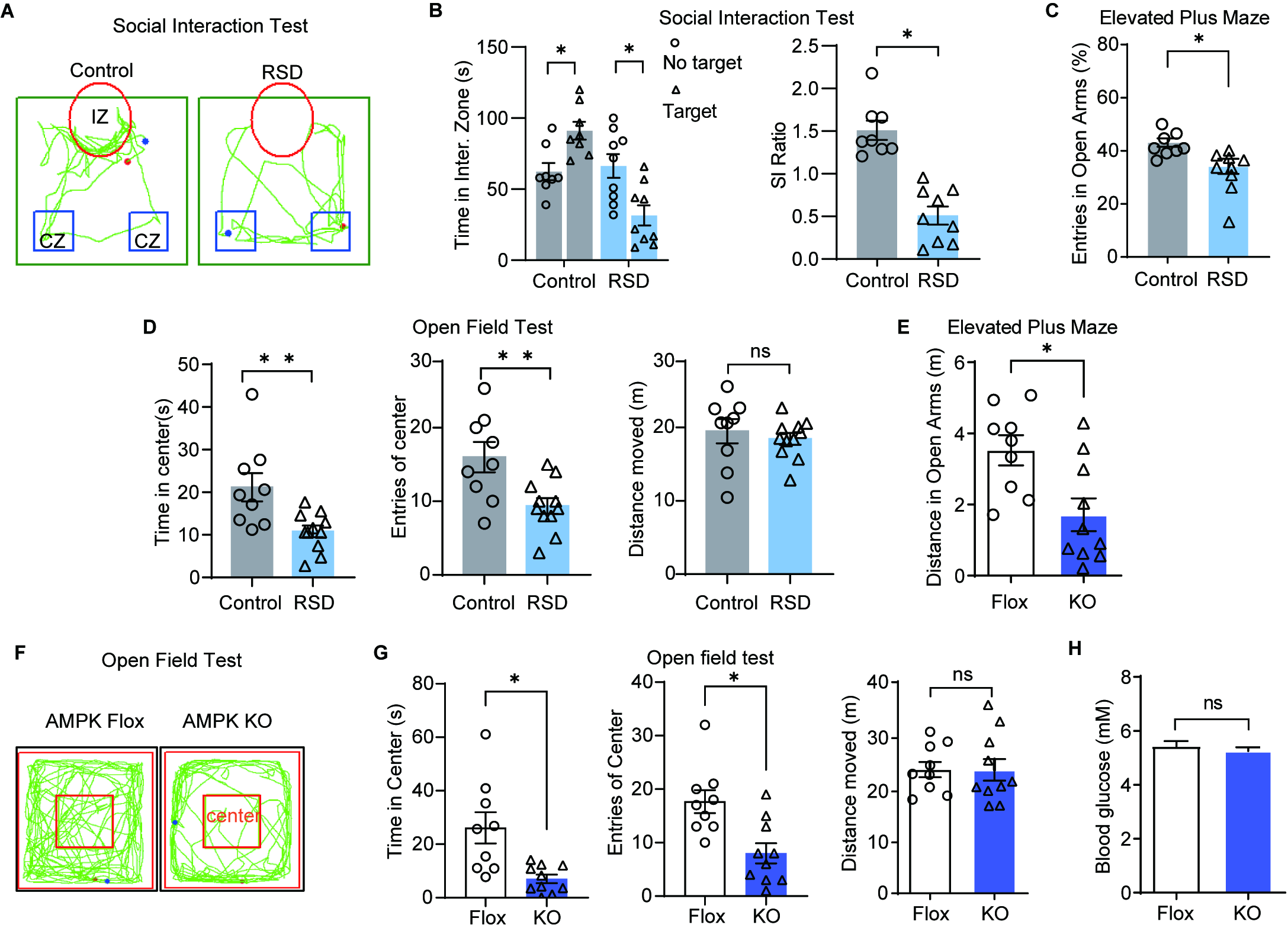

Supplement: Supplementary file 2 — Supplemental figure 1 [file 41380_2023_2283_MOESM2_ESM.tif]

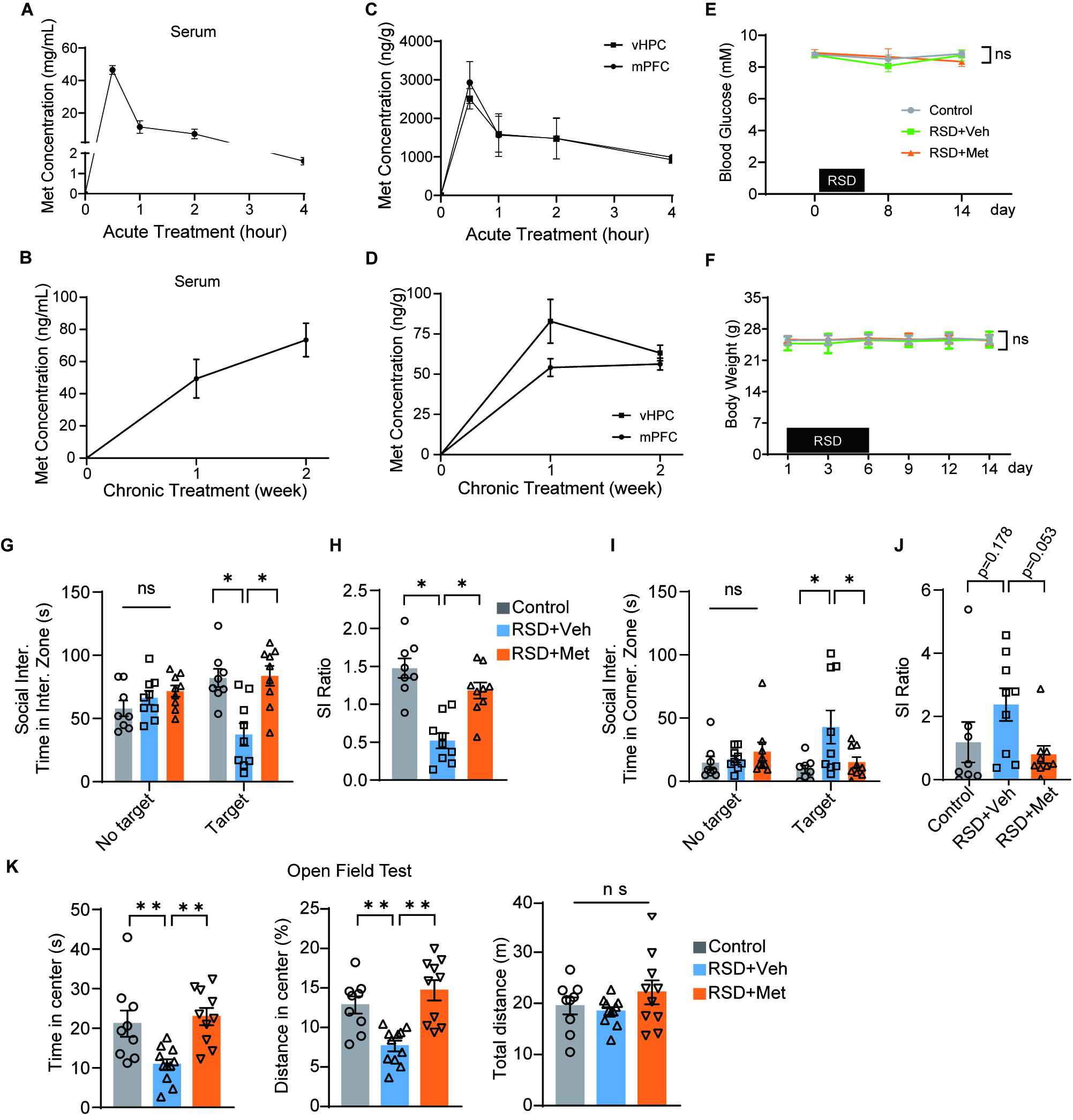

Supplement: Supplementary file 3 — Supplemental figure 2 [file 41380_2023_2283_MOESM3_ESM.tif]

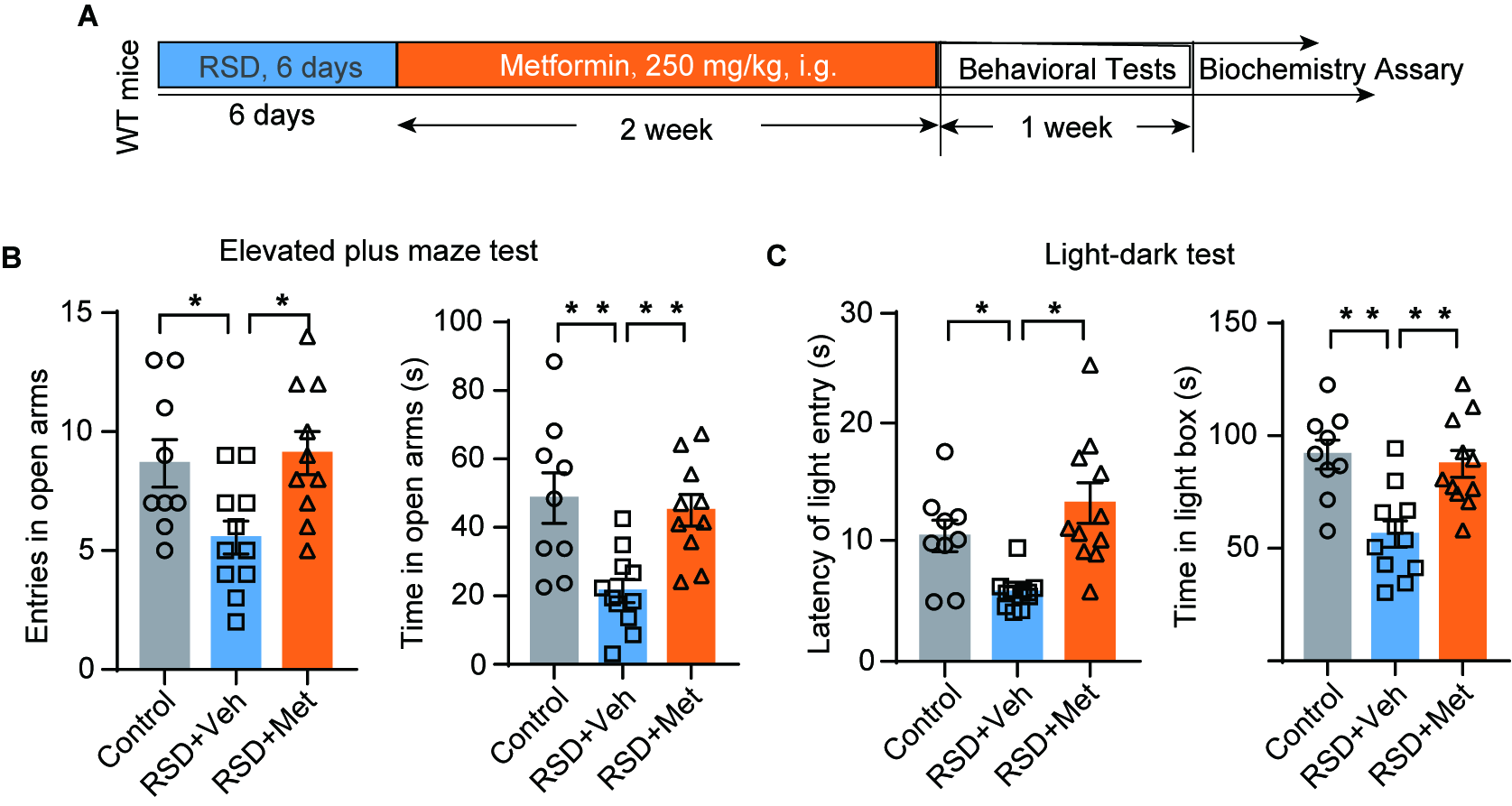

Supplement: Supplementary file 4 — Supplemental figure 3 [file 41380_2023_2283_MOESM4_ESM.tif]

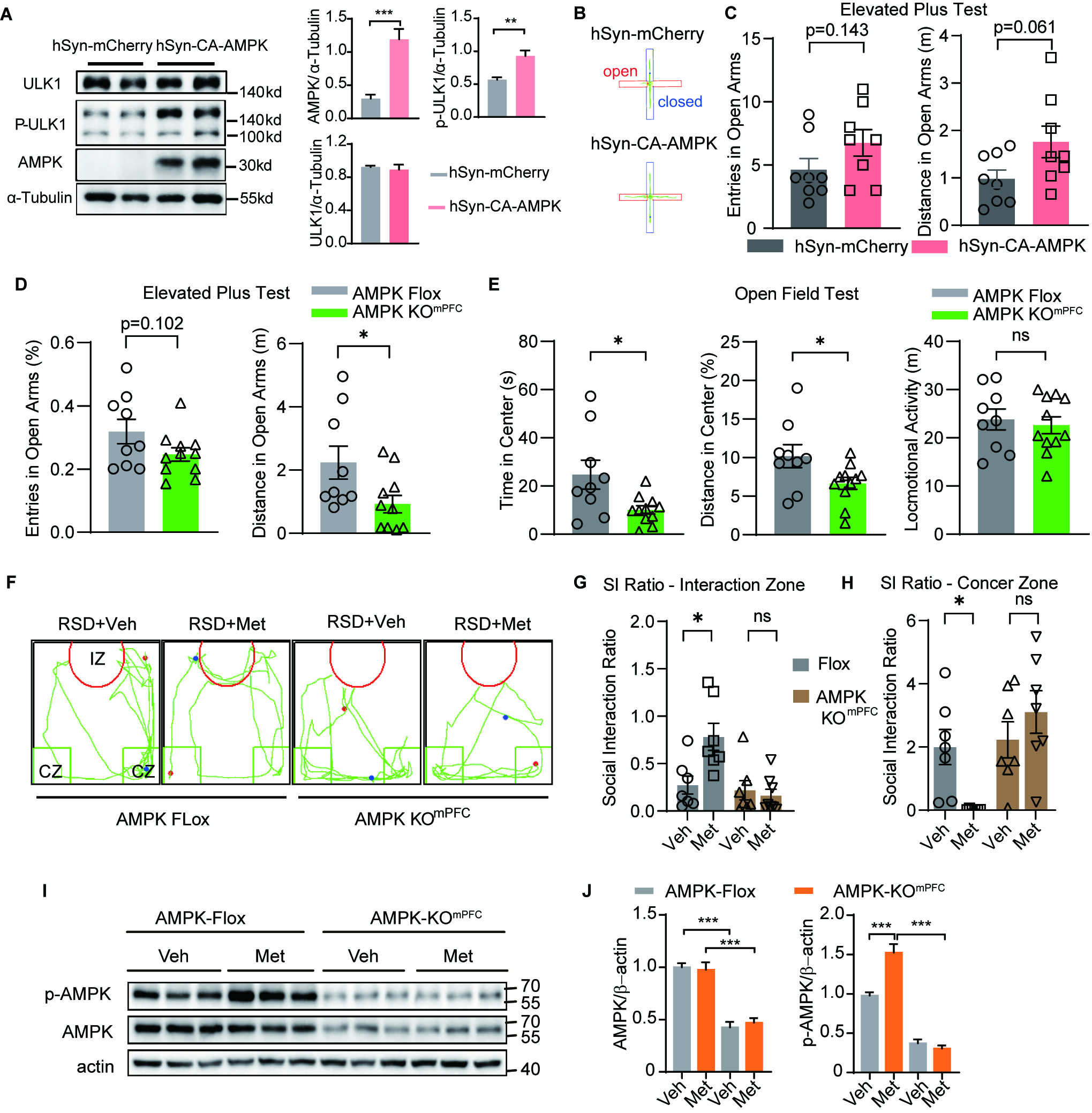

Supplement: Supplementary file 5 — Supplemental figure 4 [file 41380_2023_2283_MOESM5_ESM.tif]

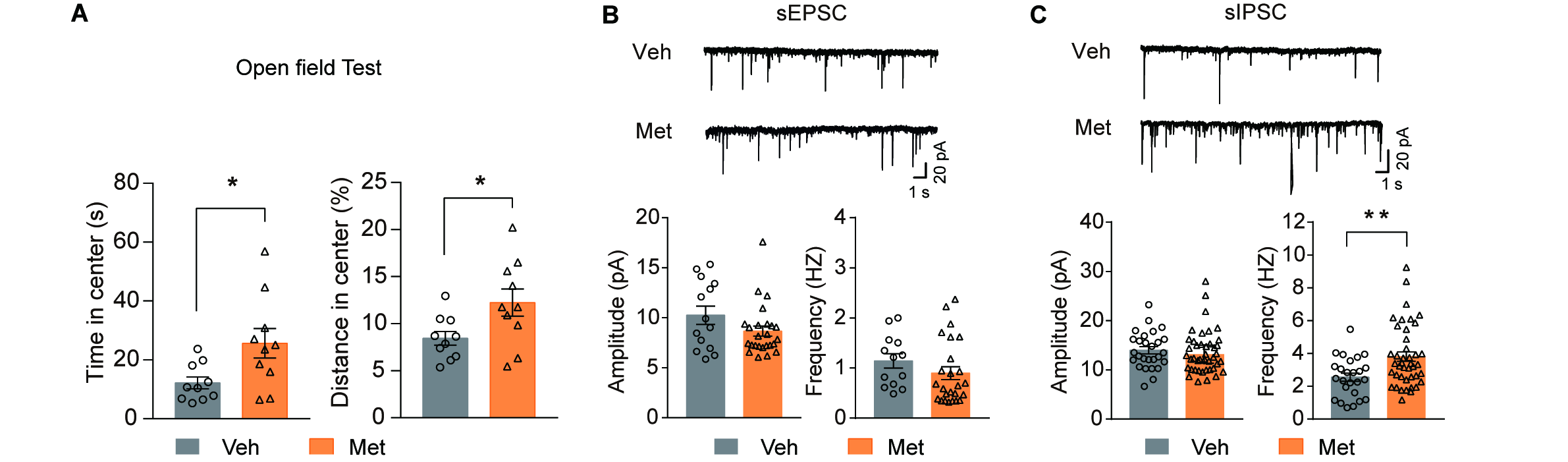

Supplement: Supplementary file 6 — Supplemental figure 5 [file 41380_2023_2283_MOESM6_ESM.tif]

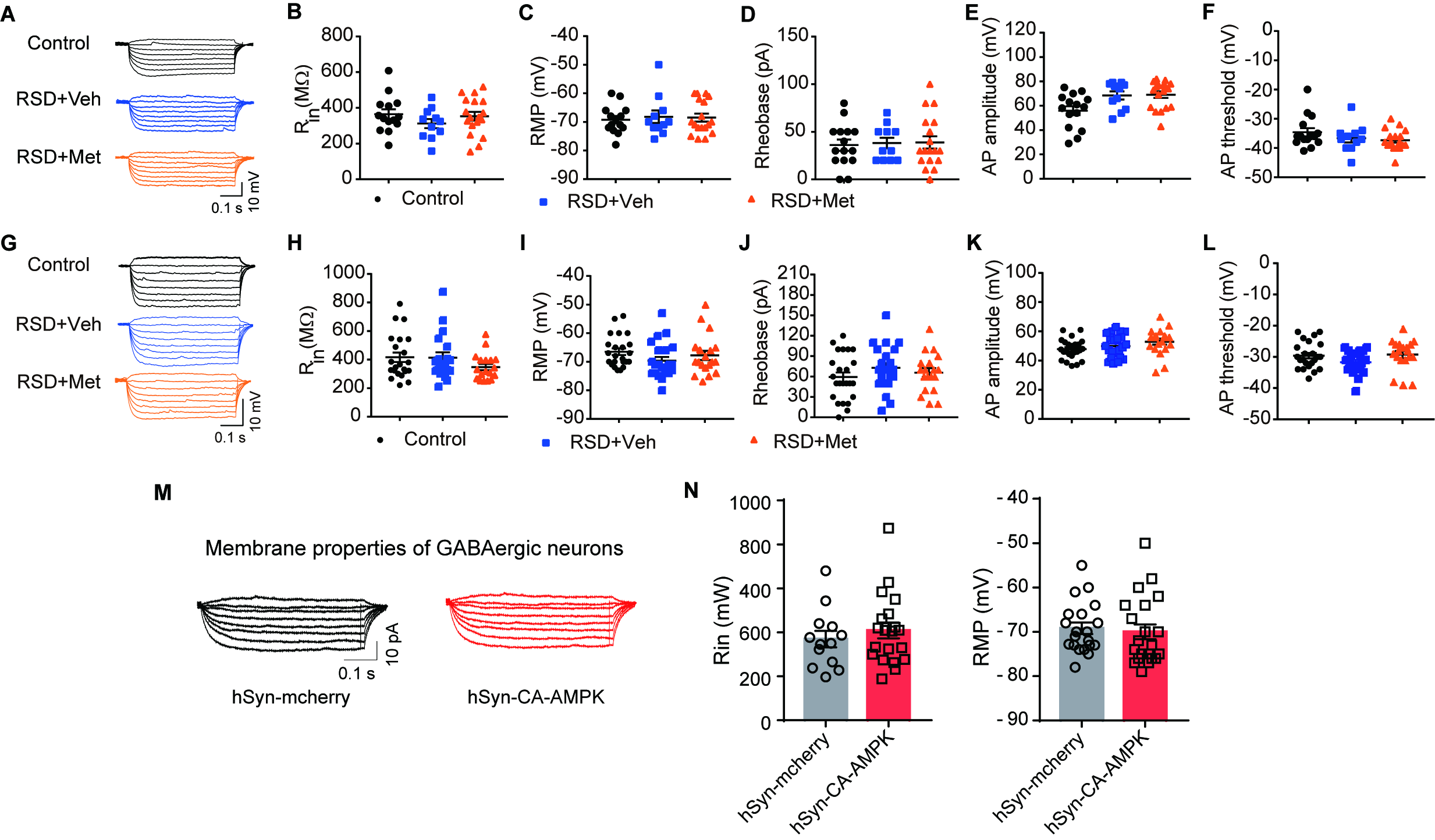

Supplement: Supplementary file 7 — Supplemental figure 6 [file 41380_2023_2283_MOESM7_ESM.tif]

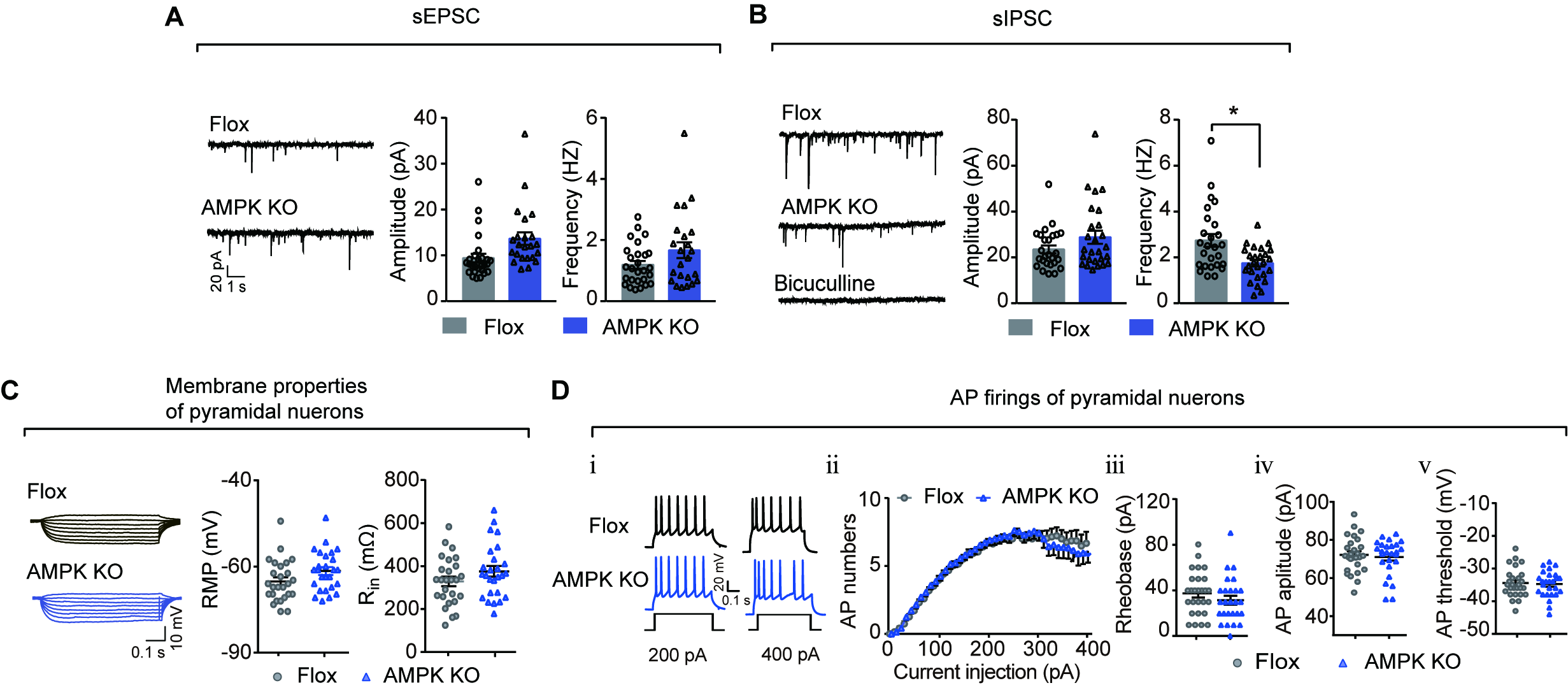

Supplement: Supplementary file 8 — Supplemental figure 7 [file 41380_2023_2283_MOESM8_ESM.tif]

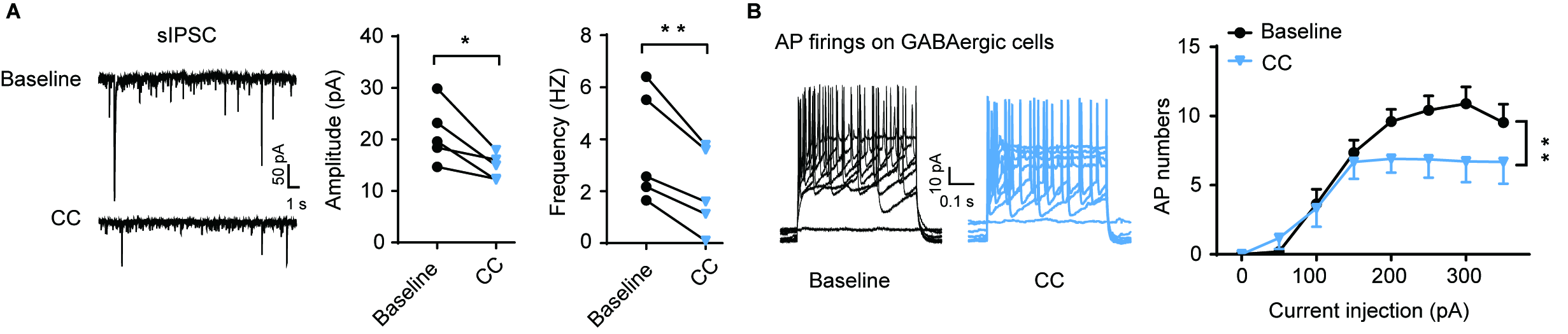

Supplement: Supplementary file 9 — Supplemental figure 8 [file 41380_2023_2283_MOESM9_ESM.tif]

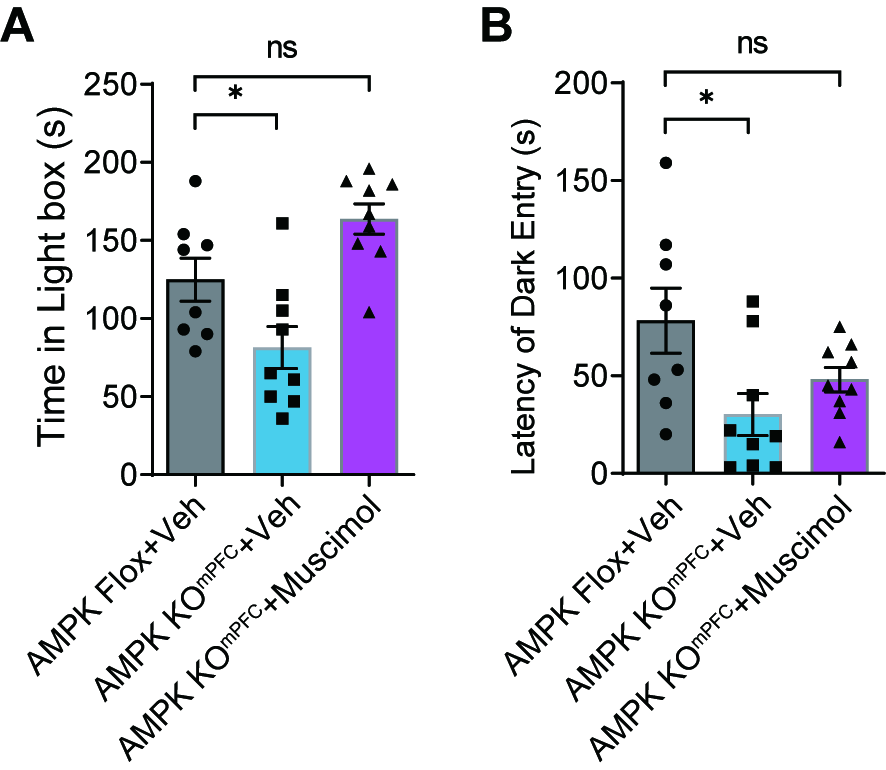

Supplement: Supplementary file 10 — Supplemental figure 9 [file 41380_2023_2283_MOESM10_ESM.tif]

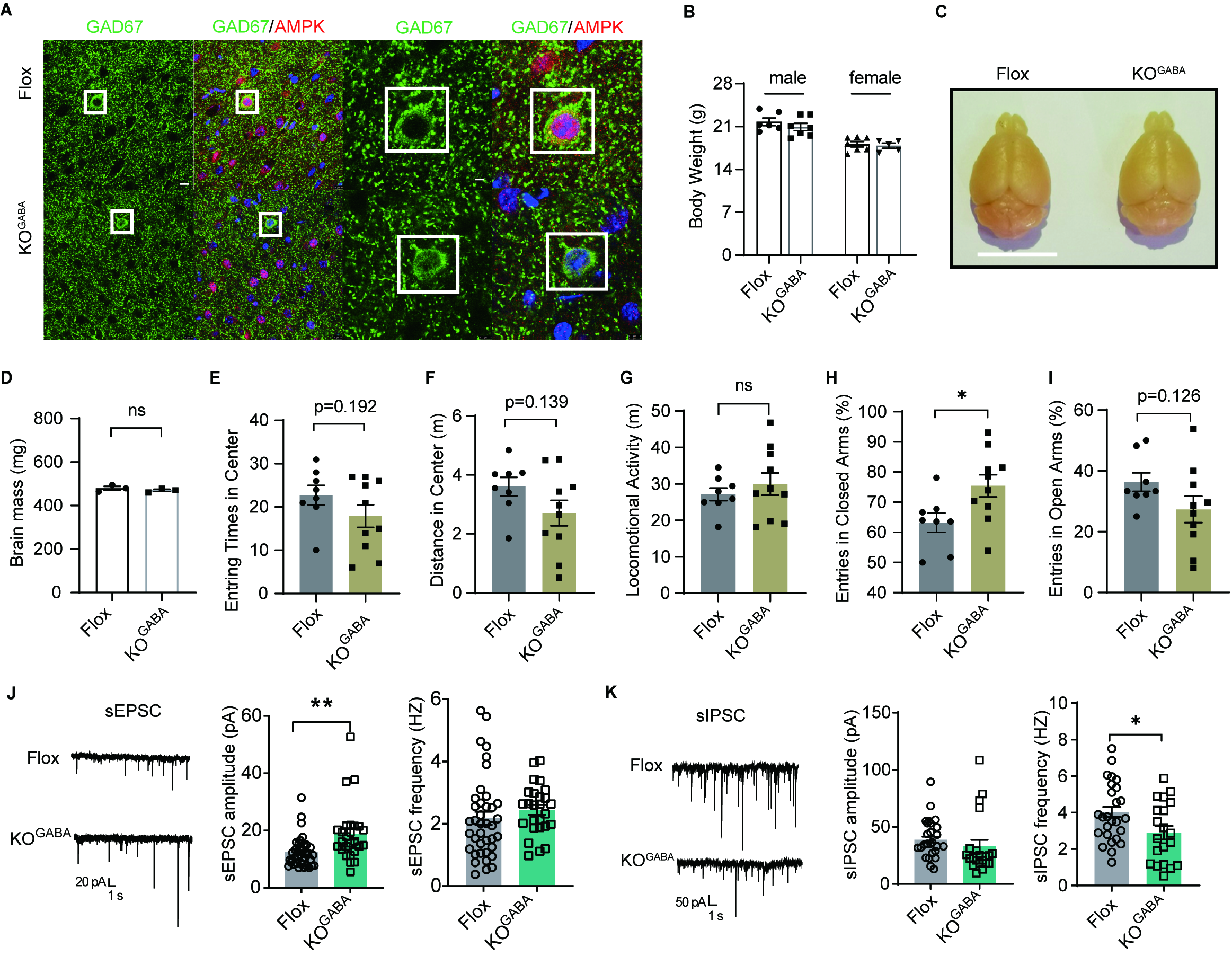

Supplement: Supplementary file 11 — Supplemental figure 10 [file 41380_2023_2283_MOESM11_ESM.tif]

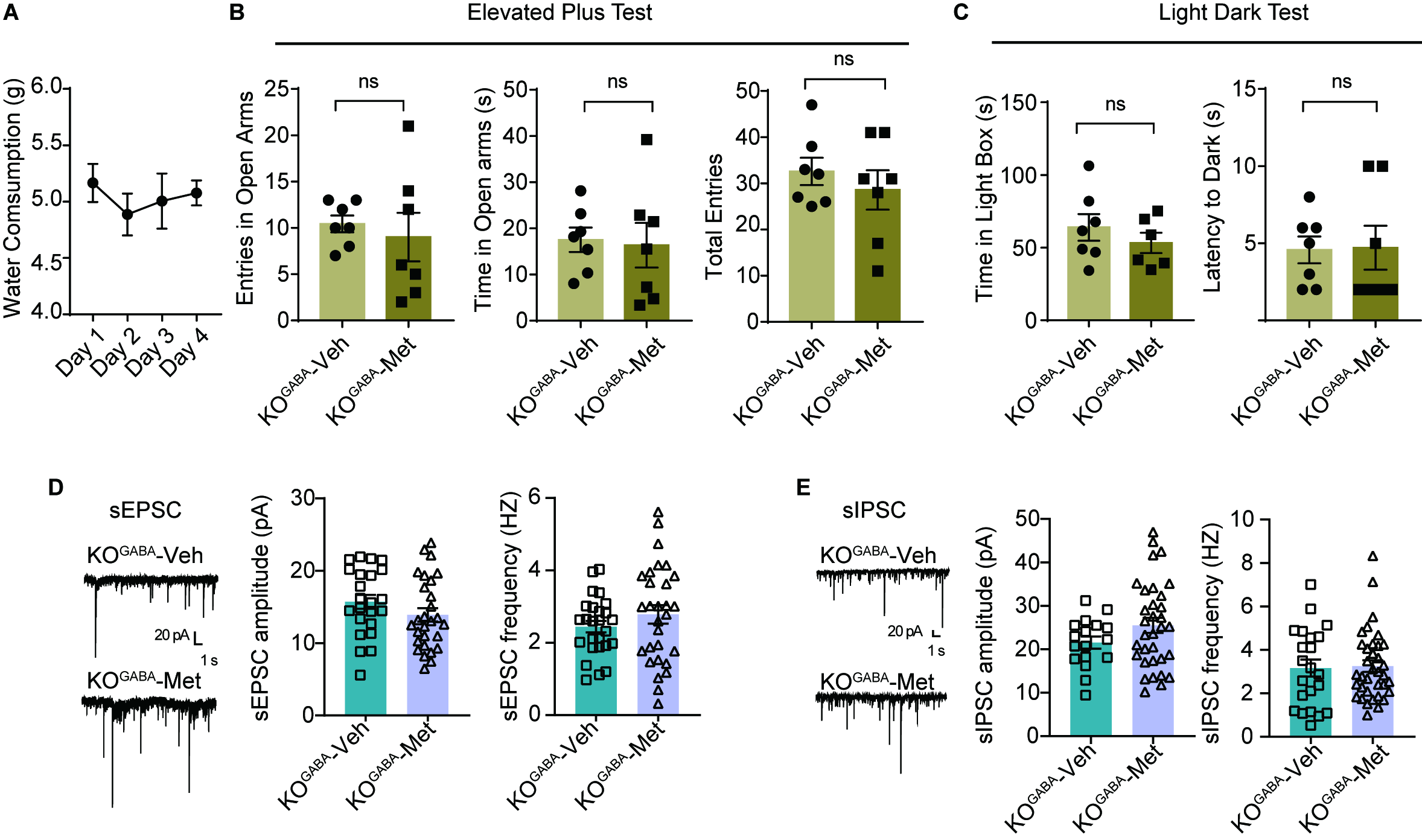

Supplement: Supplementary file 12 — Supplemental figure 11 [file 41380_2023_2283_MOESM12_ESM.tif]
