# Supplementary material for: Anxiolytic effect of antidiabetic metformin is mediated by AMPK activation in mPFC inhibitory neurons
Source: Mol Psychiatry. 2023 Oct 5;28(9):3955–65. doi: 10.1038/s41380-023-02283-w (PMC10730396; doi:10.1038/s41380-023-02283-w)
Supplement: Supplementary file 13 — Key Resources Table [file 41380_2023_2283_MOESM13_ESM.docx]

**Key Resources Table**

| **REAGENT or RESOURCE** | **SOURCE** | **IDENTIFIER** |
| --- | --- | --- |
| **Antibodies** |  |  |
| Anti-AMPKα Rabbit polyclonal antibody | Cell Signaling Technology | Cat# 2532 |
| Anti-Phospho-AMPKα (Thr172) (40H9) Rabbit monoclonal antibody | Cell Signaling Technology | Cat# 2535 |
| Anti-β-actin Mouse monoclonal antibody | Abcepta | Cat#AM1021B |
| **Bacterial and virus strains** |  |  |
| AAV2/9-CMV-Cre-EGFP | Taitool Bioscience (Shanghai) | N/A |
| AAV2/9-CMV-EGFP | Taitool Bioscience (Shanghai) | N/A |
| AAV2/8-hSyn-CA-AMPK-mCherry | Taitool Bioscience (Shanghai) | N/A |
| AAV2/8-hSyn-mCherry | Taitool Bioscience (Shanghai) | N/A |
| **Chemicals, peptides, and recombinant proteins** |  |  |
| Metformin hydrochloride | MedChemExpress | Cat#HY-17471A |
| Muscimol | BACHEM | Cat#101995 |
| CNQX | SIGMA | Cat#115066-14-3 |
| Bicuculline | TOCRIS | Cat#0130 |
| MK-801 | SIGMA | Cat#77086-22-7 |
| Compound C | MedChemExpress | Cat# HY-13418A |
| **Experimental models: Organisms/strains** |  |  |
| C57BL/6J mice | SLAC Laboratory animal, Shanghai | N/A |
| CD1 mice | SLAC Laboratory animal, Shanghai | N/A |
| Mouse: Prkaa1 loxp/loxp | Jackson Laboratory | Strain #:014141 |
| Mouse: Prkaa2 loxp/loxp | Jackson Laboratory | Strain #:014142 |
| Mouse: Nestin-Cre | Jackson Laboratory | Strain #:003771 |
| Mouse: Vagt-ires-Cre | Jackson Laboratory | Strain #:016962 |
| Mouse: GAD1-GFP | Shanghai model organisms, Shanghai | N/A |
| **Software and algorithms** |  |  |
| SuperMaze | Shanghai XinRuan Information Technology, Shanghai | http://www.softmaze.com/s02/product/2014/07/07/20971114.html |
| Mini analysis program | Synaptosoft | https://en.freedownloadmanager.org/Windows-PC/Mini-Analysis-Program.html |
| OriginPro 8.1 SR3 | PortableSoft | https://www.originlab.com/ |
| pCLAMP | Molecular Devices, LLC. | https://support.moleculardevices.com/s/article/Axon-pCLAMP-11-Electrophysiology-Data-Acquisition-Analysis-Software-Download-Page |
| Prism | GraphPad Software | https://www.graphpad.com/scientific-software/prism |
| ImageJ | National Institutes of Health | https://imagej.nih.gov/ij/index.html |
